# Potentiation of fentanyl-induced respiratory depression by alcohol is not fully reversed by naloxone

**DOI:** 10.1172/jci.insight.198059

**Published:** 2026-02-03

**Authors:** Emma V. Frye, Lyndsay E. Hastings, Aniah N. Matthews, Adriana Gregory-Flores, Janaina C.M. Vendruscolo, Lindsay A. Kryszak, Shelley N. Jackson, Aidan J. Hampson, Nora D. Volkow, Leandro F. Vendruscolo, Renata C.N. Marchette, George F. Koob

**Affiliations:** 1 Neurobiology of Addiction Section and; 2 Translational Analytical Core, National Institute on Drug Abuse, Intramural Research Program, NIH, Baltimore, Maryland, USA.; 3 Division of Pharmacotherapeutic Development, National Institute on Drug Abuse, Rockville, Maryland, USA.; 4 Laboratory for Neuroimaging, National Institute on Alcohol Abuse and Alcoholism, Intramural Research Program, and; 5 Stress and Addiction Neuroscience Unit, National Institute on Drug Abuse and National Institute on Alcohol Abuse and Alcoholism, Intramural Research Programs, NIH, Baltimore, Maryland, USA.

**Keywords:** Neuroscience, Public Health, Addiction, Pharmacology, Respiration

## Abstract

The high frequency of opioid overdose deaths often involves co-use of alcohol, which is reported in approximately 30% of fentanyl fatalities. Both substances depress respiratory function, and their combined effects can be lethal. The present study investigated physiological parameters of respiratory-depressant effects of fentanyl when coadministered with alcohol and their sensitivity to naloxone reversal using whole-body plethysmography in male and female Long-Evans rats. Administration of a high, sedative-like dose of alcohol alone or fentanyl alone resulted in no mortality, but fentanyl plus alcohol led to mortality rates of 42% and 33% in females and males, respectively. The fentanyl+alcohol combination reduced minute ventilation and increased apneic pauses compared with either drug alone. Lower, binge-like alcohol doses when combined with fentanyl also amplified respiratory depression. Pretreatment with naloxone did not fully restore normal respiration. Naloxone administered after fentanyl+alcohol transiently reversed the decrease in minute ventilation but did not reverse apneic pauses. Fentanyl-dependent rats were partially tolerant to fentanyl- and fentanyl+alcohol–induced respiratory depression, but alcohol-dependent rats exhibited sensitization to alcohol- and fentanyl+alcohol–induced apnea. These findings highlight physiological parameters of severe respiratory risks with fentanyl+alcohol co-use, which are inadequately reversed by naloxone, underscoring the need for targeted strategies to manage opioid+alcohol overdoses.

## Abstract 2

Overdoses resulting from the combination of fentanyl and alcohol lead to severe apnea that is not fully rescued by naloxone.

## Graphical abstract

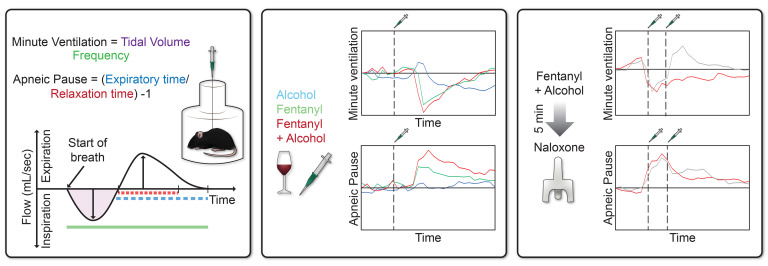

## Introduction

The number of drug overdose deaths that involve opioids in the United States started to decrease in 2023, but still 54,745 fatalities were recorded in 2024 (1). Fentanyl is associated with 88% of current opioid overdose fatalities (2), but other drugs, including alcohol, are frequently co-involved (3). Alcohol is present in 30% of fatalities that are attributed to heroin and fentanyl (4), but high doses of alcohol alone can also lead to fatal respiratory depression (5–7).

The higher risk of overdoses when opioids are combined with alcohol has been well documented (8, 9), but the mechanisms have not been well characterized. Overdose deaths that involve opioid plus alcohol combinations doubled between 2000 and 2019 (10), which highlights the urgency to investigate mechanisms of lethality caused by their combination to guide the development of effective reversal interventions.

Opioid overdose deaths are primarily attributed to respiratory depression, an effect that is mediated by μ-opioid receptors (MORs) in brainstem respiratory nuclei, which modulate respiratory rhythms in response to sensory feedback from carotid bodies and respiratory muscles (11). The mechanism by which alcohol induces respiratory depression is unclear, but early reports suggested the involvement of endogenous opioid signaling pathways (12, 13).

Naloxone, a preferential MOR antagonist, is the gold-standard treatment to reverse opioid-induced respiratory depression. However, its efficacy in reversing the depressant effects of fentanyl+alcohol is not well characterized. Understanding such interactions is foundational for the development of better reversal treatments. We hypothesized that fentanyl+alcohol would have additive respiratory-depressant effects, and that naloxone would be insufficient to completely reverse it.

## Results

### Additive effects of 25 μg/kg fentanyl and 1.18 g/kg alcohol on minute ventilation and apneic pauses

**Figure 1:**
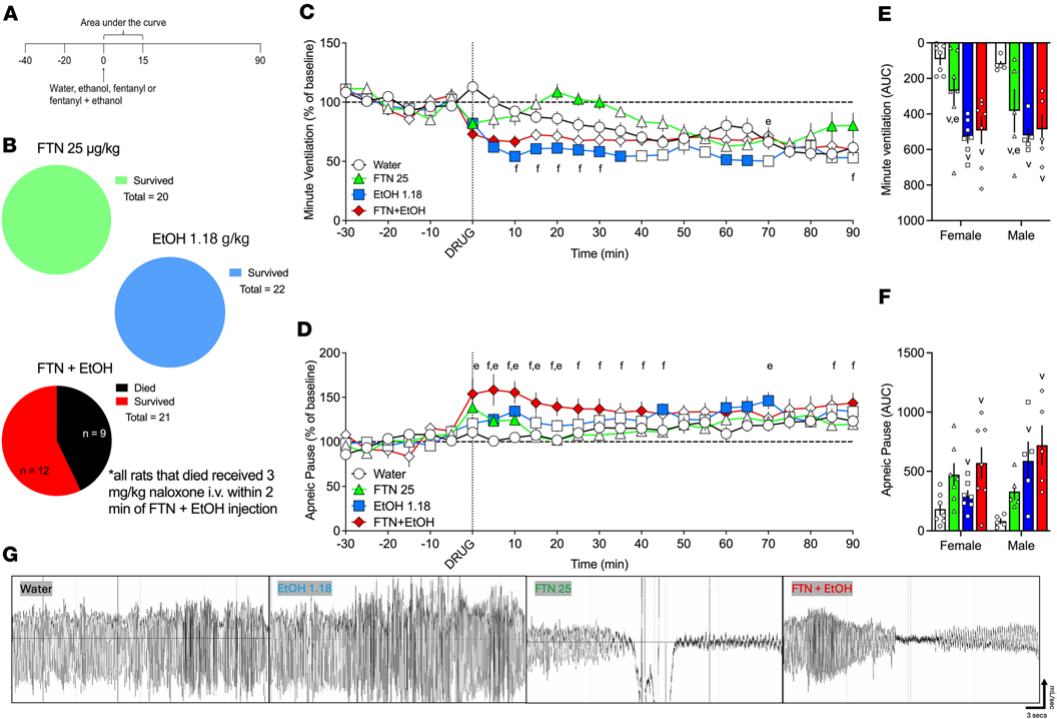
Effects of 25 μg/kg fentanyl and 1.18 g/kg alcohol alone and combined on minute ventilation and apneic pauses. Rats received i.v. infusions of sterile water (5 mL/kg), fentanyl (25 μg/kg, 5 mL/kg), alcohol (1.18 g/kg, 30% vol/vol, 5 mL/kg), or a fentanyl+alcohol combination (25 μg/kg and 1.18 g/kg, respectively, 5 mL/kg) in a within-subjects Latin square design with each test separated by 1 week. (**A**) Timeline of each test. (**B**) Mortality for each drug. Data from the 12 rats that completed the experiment are shown in **C**–**F**. (**C**) Alcohol and fentanyl, alone and combined, decreased minute ventilation in a time-dependent manner. (**D**) Alcohol and fentanyl alone and combined increased apneic pauses in a time-dependent manner. The data are expressed as the mean ± SEM and were analyzed by 2-way RM-ANOVA followed by Duncan’s post hoc test. Filled symbols are different from water. ^f^Combination different from fentanyl; ^e^combination different from alcohol (*P* < 0.05). (**E**) AUC of the first 15 min after infusion for minute ventilation. (**F**) AUC of the first 15 min after infusion for apneic pauses. The data are expressed as the mean ± SEM and were analyzed by 2-way RM-ANOVA followed by Šidák’s post hoc test when appropriate. Main effect of treatment: ^v^*P* < 0.05, compared with water; ^e^*P* < 0.05, compared with alcohol. *n* = 7 females, 5 males. (**G**) Representative raw plethysmography traces.

The first experiment sought to investigate whether a high, sedative-like dose of alcohol has additive effects to fentanyl in ventilatory parameters. The combination of 25 μg/kg fentanyl and the high dose of 1.18 g/kg alcohol resulted in high mortality (41.7% females, 33.3% males; Figure 1B and Supplemental Figure 2; supplemental material available online with this article; https://doi.org/10.1172/jci.insight.198059DS1), whereas neither alcohol nor fentanyl alone produced mortality. Figure 1 shows data from rats that completed all tests.

#### Analysis over time.

The repeated-measures ANOVA (RM-ANOVA) for minute ventilation showed a main effect of time (*F*_18,180_ = 6.8939, *P* < 0.00001) and of treatment (*F*_3,30_ = 11.946, *P* = 0.00003) and a treatment × time interaction (*F*_54,540_ = 2.782, *P* < 0.0001; Figure 1C). Fentanyl decreased minute ventilation immediately upon injection (0 min) and increased ventilation at 20–30 and 85–90 min after infusion compared with vehicle. Alcohol decreased minute ventilation at 0–35 and 60–65 min after infusion, and fentanyl+alcohol decreased minute ventilation at 0–10 min after infusion compared with vehicle and at 10–30 min after infusion compared with fentanyl alone. There was no significant effect of sex, no sex × treatment interaction, no sex × time interaction, and no sex × treatment × time interaction.

The RM-ANOVA for apneic pause showed a main effect of time (*F*_18,180_ = 2.6952, *P* = 0.0004) and of treatment (*F*_3,30_ = 3.3695, *P* = 0.031) and a significant treatment × time interaction (*F*_54,54_ = 2.0224, *P* < 0.0001; Figure 1D). Fentanyl increased apneic pauses at 0–10 min after infusion, alcohol at 5–10, 45, and 60–70 min after infusion, and fentanyl+alcohol at 0–30, 40, 60, and 90 min after infusion compared with vehicle. Fentanyl+alcohol also increased apneic pauses at 0–20 and 70 min after infusion compared with alcohol alone, and at 5–45, 85, and 90 min after infusion compared with fentanyl alone. There was no significant effect of sex, no sex × treatment interaction, no sex × time interaction, and no sex × treatment × time interaction.

#### AUC.

The AUC of minute ventilation for the first 15 min after infusion showed a main effect of treatment (*F*_3,40_ = 15.05, *P* < 0.0001). Fentanyl alone (*P* = 0.018), alcohol alone (*P* < 0.00001), and fentanyl+alcohol (*P* < 0.00001) induced a greater decrease in minute ventilation than vehicle (Figure 1E). Alcohol alone had a greater effect than fentanyl alone (*P* = 0.0423). Fentanyl+alcohol had similar effects to alcohol (*P* = 0.99) and fentanyl alone (*P* = 0.13). There was no effect of sex and no sex × treatment interaction.

The AUC of apneic pause for the first 15 min after infusion showed a main effect of treatment (*F*_3,40_ = 8.719, *P* = 0.0001). Šidák’s post hoc test showed that alcohol alone (*P* = 0.024) and fentanyl+alcohol (*P* < 0.00001) induced a greater increase in apneic pauses than vehicle, but fentanyl alone did not (*P* = 0.065; Figure 1F), whereas increases in apneic pauses between fentanyl+alcohol and alcohol did not differ (*P* = 0.27). There was no effect of sex and no sex × treatment interaction.

The AUCs for the other 9 parameters are shown in Table 1. Fentanyl+alcohol had additive effects on tidal volume, inspiratory time, peak expiratory flow, and end-inspiratory pause compared with fentanyl and alcohol alone. Altogether, these data indicate an additive effect of a sedative-like dose of alcohol with fentanyl on ventilatory parameters.

### Additive effects of 25 μg/kg fentanyl and 0.59 g/kg alcohol on minute ventilation and apneic pauses

The second experiment sought to investigate whether a binge-like dose of alcohol has additive effects to fentanyl in ventilatory parameters.

#### Analysis over time.

**Figure 2:**
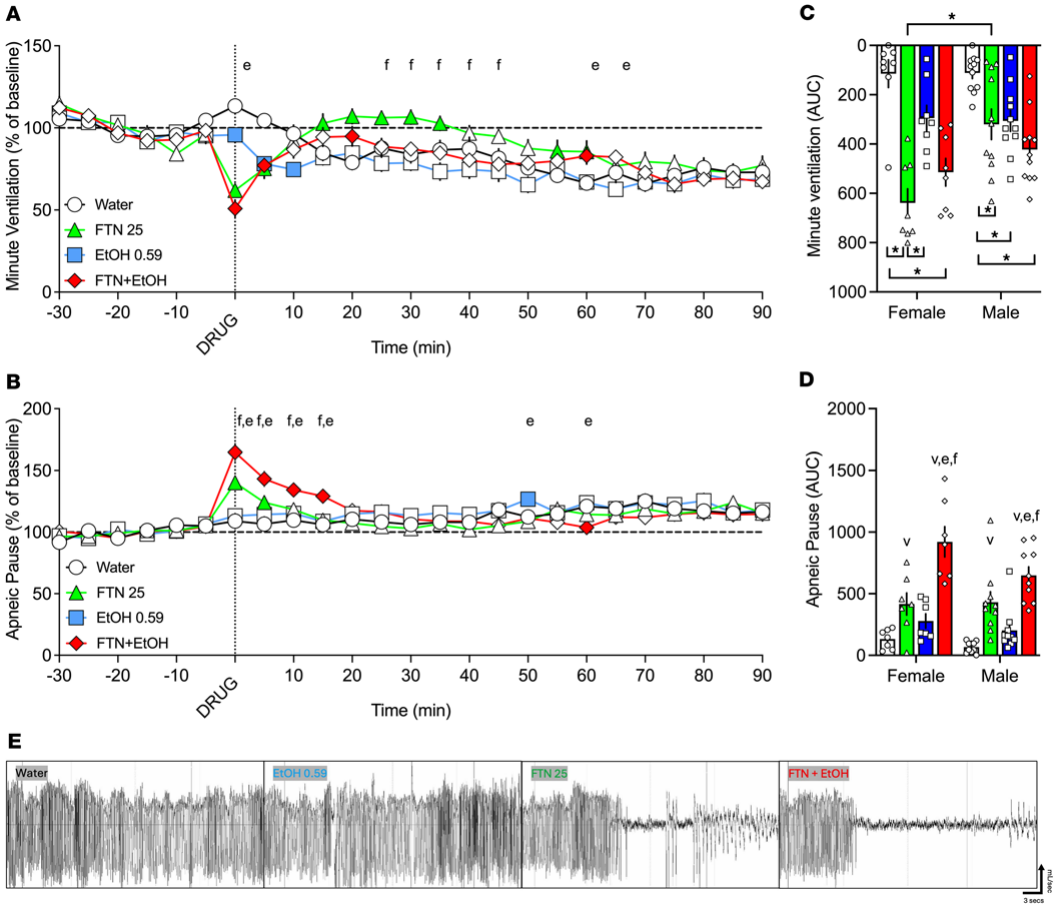
Effects of 25 μg/kg fentanyl and 0.59 g/kg alcohol alone and combined on minute ventilation and apneic pauses. Rats received i.v. infusions of sterile water (2.5 mL/kg), fentanyl (25 μg/kg, 2.5 mL/kg), alcohol (0.59 g/kg, 30% vol/vol, 2.5 mL/kg), or a fentanyl+alcohol combination (25 μg/kg and 0.59 g/kg, respectively, 2.5 mL/kg) in a within-subjects Latin square design with each test separated by 1 week. (**A**) Alcohol, fentanyl, and fentanyl+alcohol decreased minute ventilation in a time-dependent manner. (**B**) Alcohol, fentanyl, and fentanyl+alcohol increased apneic pauses in a time-dependent manner. The data are expressed as the mean ± SEM and were analyzed by 2-way RM-ANOVA followed by Duncan’s post hoc test when appropriate. Filled symbols are different from water. ^f^Combination different from fentanyl; ^e^combination different from alcohol (*P* < 0.05). (**C**) AUC of the first 15 min after infusion for minute ventilation. *P < 0.05, drug × sex interactions. (**D**) AUC of the first 15 min after infusion for apneic pauses. The data are expressed as the mean ± SEM and were analyzed by 2-way RM-ANOVA followed by Šidák’s post hoc test when appropriate. Main treatment effect: ^v^*P* < 0.05, compared with water; ^f^*P* < 0.05, compared with fentanyl; ^e^*P* < 0.05, compared with alcohol. *n* = 7–8 females, 10–11 males. (**E**) Representative raw plethysmography traces.

The RM-ANOVA for minute ventilation showed a main effect of time (*F*_18,324_ = 10.064, *P* < 0.00001) and of treatment (*F*_3,54_ = 6.512, *P* = 0.0011) and a significant treatment × time interaction (*F*_54,972_ = 5.910, *P* < 0.0001). Fentanyl alone decreased minute ventilation at 0 and 5 min and increased ventilation at 15–35 and 55–60 min after infusion (*P* < 0.05); alcohol alone decreased minute ventilation at 0–10 min after infusion (*P* < 0.05), whereas fentanyl+alcohol decreased minute ventilation at 0–10, 20, and 60 min after infusion compared with vehicle. Fentanyl+alcohol decreased minute ventilation at 25–45 min after infusion compared with fentanyl alone and at 0, 20, and 60 min after infusion compared with alcohol alone (*P* < 0.05; Figure 2A). There was no significant effect of sex, no sex × treatment interaction, no sex × time interaction, and no sex × treatment × time interaction.

The RM-ANOVA for apneic pauses showed a main effect of time (*F*_18,324_ = 5.920, *P* <0.0001), no main effect of treatment (*F*_3,54_=1.115, *P* = 0.351), and a significant treatment × time interaction (*F*_54,972_ = 5.396, *P* < 0.0001; Figure 2B). Fentanyl increased apneic pauses at 0–5 min after infusion (*P* < 0.05), alcohol at 50 min after infusion (*P* < 0.05), and fentanyl+alcohol at 0–15 and 60 min after infusion compared with vehicle. Fentanyl+alcohol also increased apneic pauses at 0–15 min after infusion compared with fentanyl alone, and at 0–15, 50, and 60 min after infusion compared with alcohol alone (*P* < 0.05). There was no main effect of sex, no sex × treatment interaction, and no sex × treatment × time interaction. There was a significant sex × time interaction (*F*_18,324_ = 2.712, *P* = 0.0002), with males and females differing at 0 and 5 min.

#### AUC.

For minute ventilation the AUC for the first 15 min after infusion showed a main effect of treatment (*F*_3,68_ = 23.81, *P* < 0.0001; Figure 2C). There was a significant effect of sex (*F*_1,68_ = 8.133, *P* = 0.0058; females > males) and a significant sex × treatment interaction (*F*_3,68_ = 4.672, *P* = 0.0050). Females that received fentanyl exhibited a greater decrease in minute ventilation than males (*P* = 0.0001). In females, fentanyl (*P* < 0.0001) and fentanyl+alcohol (*P* < 0.0001) but not alcohol (*P* = 0.217) induced a greater decrease than vehicle. In males, fentanyl (*P* = 0.024), alcohol (*P* = 0.044), and fentanyl+alcohol (*P* = 0.0001) produced a greater decrease than vehicle.

For apneic pauses, the AUC for the first 15 min after infusion showed a main effect of treatment (*F*_3,60_ = 34.26, *P* < 0.0001). Fentanyl (*P* = 0.0002) and fentanyl+alcohol (*P* < 0.00001) caused a greater increase than vehicle (Figure 2D), and fentanyl+alcohol induced a greater effect than fentanyl (*P* < 0.0001) and alcohol (*P* < 0.0001). There was a trend toward an effect of sex (*F*_1,60_ = 3.900, *P* = 0.053; females > males) but no sex × treatment interaction (*F*_3,60_ = 1.456, *P* = 0.236).

The AUCs for the other 9 parameters are shown in Table 2. All treatments increased AUC of all 9 parameters. Females had a more pronounced effect of fentanyl on inspiratory time, peak expiratory flow, and end-expiratory pause and a more pronounced effect of fentanyl+alcohol on tidal volume and a blunted effect on inspiratory time compared with males. Altogether, these data indicate an additive effect of a binge-like dose of alcohol with fentanyl on ventilatory parameters.

### A low dose of fentanyl (3.125 μg/kg) did not have additive effects with 0.59 g/kg alcohol

The third experiment investigated whether a binge-like dose of alcohol had additive effects with a low dose of fentanyl in ventilatory parameters (Supplemental Figure 4 and Supplemental Table 1). Altogether, the data indicate no additive effect of a binge-like dose of alcohol with a low dose of fentanyl on ventilatory parameters.

### Naloxone injected 5 min after fentanyl+alcohol administration reversed the effect of fentanyl+alcohol (25 μg/kg + 0.59 g/kg) on minute ventilation but not on apneic pauses

The fourth experiment investigated whether naloxone could rescue or prevent the effects of fentanyl+alcohol on ventilatory parameters.

#### Naloxone (100 μg/kg): analysis over time.

**Figure 3:**
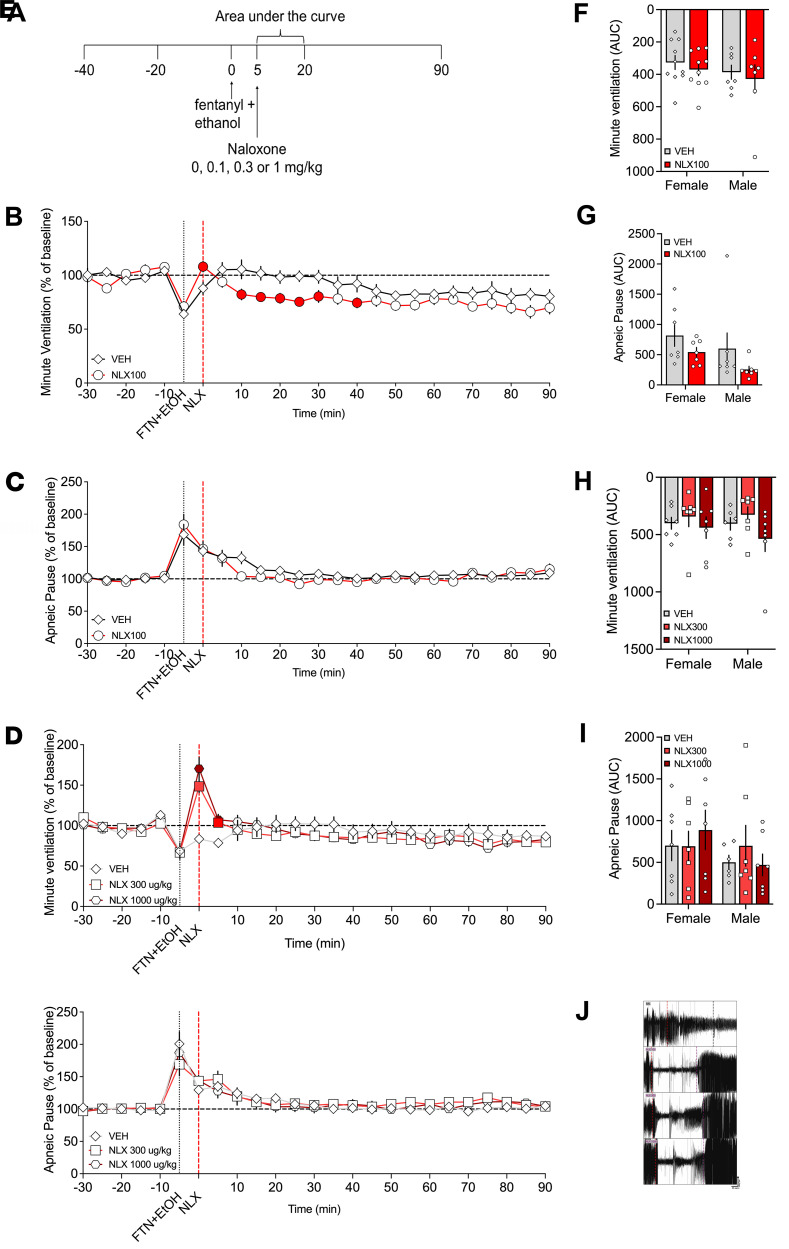
Effect of naloxone on respiratory depression induced by fentanyl+alcohol (25 μg/kg + 0.59 g/kg). Rats received an i.v. infusion of a fentanyl+alcohol combination (25 μg/kg and 0.59 g/kg, 2.5 mL/kg), followed by an injection of naloxone (0, 100, 300, and 1,000 μg/kg) 5 min later. (**A**)Timeline of each test. (**B**) Naloxone (100 μg/kg) transiently reversed the fentanyl+alcohol–induced decrease in minute ventilation. (**C**) Naloxone (100 μg/kg) did not change the fentanyl+alcohol–induced increase in apneic pauses. (**D**) Naloxone (300 and 1,000 μg/kg) reversed fentanyl+alcohol–induced decreases in minute ventilation. (**E**) Naloxone (300 and 1,000 μg/kg) did not change the fentanyl+alcohol–induced increase in apneic pauses. The data are expressed as the mean ± SEM and were analyzed by 2-way RM-ANOVA followed by Duncan’s post hoc test when appropriate. (**F**) AUC of the first 15 min after naloxone infusion for minute ventilation. (**G**) AUC of the first 15 min after naloxone infusion for apneic pauses. (**H**) AUC of the first 15 min after naloxone infusion for minute ventilation. (**I**) AUC of the first 15 min after naloxone infusion for apneic pauses. The data are expressed as the mean ± SEM and were analyzed by 2-way RM-ANOVA followed by Šidák’s post hoc test when appropriate. *n* = 7–10 females, 7 males. (**J**) Representative raw plethysmography traces.

For minute ventilation, the 2-way RM-ANOVA revealed a main effect of time (*F*_18,270_ = 4.790, *P* < 0.0001), a main effect of naloxone treatment (*F*_1,15_ = 8.683, *P* = 0.010), and a treatment × time interaction (*F*_18,270_ = 1.860, *P* = 0.019). Naloxone restored minute ventilation upon injection at 0 min, but at 10–30 and 40 min, ventilation was inhibited to a greater degree when naloxone was present than in its absence compared with saline (Figure 3B). For apneic pauses, the 2-way RM-ANOVA revealed a main effect of time (*F*_18,23_ = 7.303, *P* < 0.0001), no main effect of treatment (naloxone) (*F*_1,13_ = 0.862, *P* = 0.370), but no treatment × time interaction (*F*_18,234_ = 0.997, *P* = 0.4645; Figure 3C). There was no effect of sex, no sex × drug interaction, and no sex × drug × time interaction for either measure.

#### Naloxone (100 μg/kg): AUC.

For AUC of minute ventilation, the 2-way RM-ANOVA revealed no main effect of sex (*F*_1,15_ = 1.272, *P* = 0.28) nor of naloxone dose (*F*_1,15_ = 0.6353, *P* = 0.44) and no sex × dose interaction (*F*_1,15_ = 0.0004, *P* = 0.9841; Figure 3F). For AUC of apneic pause, the 2-way RM-ANOVA revealed no main effect of sex (*F*_1,12_ = 2.030, *P* = 0.18) nor of naloxone dose (*F*_1,12_ = 4.210, *P* = 0.06) and no sex × dose interaction (*F*_1,12_ = 0.057, *P* = 0.81; Figure 3G).

The AUCs for the other 9 parameters are shown in Table 3. Treatment with naloxone 100 μg/kg blunted the effects of fentanyl+alcohol on frequency of breathing, inspiratory time, peak inspiratory flow, and end-expiratory pause, while it potentiated its effects on tidal volume, peak expiratory flow, and end-inspiratory pause.

#### Naloxone (300 and 1,000 μg/kg): analysis over time.

For minute ventilation with the higher naloxone doses, the 2-way RM-ANOVA revealed a main effect of time (*F*_18,216_ = 17.8438, *P* < 0.0001), no main effect of treatment (naloxone) (*F*_2,2_ = 0.0720, *P* = 0.9308), and a treatment × time interaction (*F*_36,432_ = 7.4079, *P* < 0.0001). Naloxone at 300 and 1,000 μg/kg increased minute ventilation upon injection at 0–5 min compared with saline (Figure 3D). There was no effect of sex, no sex × drug interaction, and no sex × drug × time interaction.

For apneic pauses, the 2-way RM-ANOVA revealed a main effect of time (*F*_18,216_ = 11.118, *P* < 0.0001), no main effect of treatment (naloxone) (*F*_2,24_ = 0.397, *P* = 0.6765), and no treatment × time interaction (*F*_36,432_ = 1.374, *P* = 0.0777; Figure 3E). There was no effect of sex or sex × dose × time interaction, but there was a significant sex × naloxone dose interaction (*F*_2,24_ = 3.582, *P* = 0.044). Females had higher apneic pauses after 1,000 μg/kg naloxone than males (*P* = 0.022).

#### Naloxone (300 and 1,000 μg/kg): AUC.

The 2-way RM-ANOVA of the minute ventilation AUC did not reveal a main effect of sex (*F*_1,35_ = 0.184, *P* = 0.67), a main effect of naloxone dose (*F*_2,35_ = 1.781, *P* = 0.18), or a sex × dose interaction (*F*_2,35_ = 0.273, *P* = 0.76; Figure 3H). The 2-way RM-ANOVA of the apneic pause AUC did not reveal a main effect of sex (*F*_1,12_ = 1.135, *P* = 0.31), a main effect of naloxone dose (*F*_2,23_ = 0.268, *P* = 0.77), or a sex × dose interaction (*F*_2,23_ = 0.991, *P* = 0.39; Figure 3I).

The AUCs for the other 9 parameters are shown in Table 4. Naloxone at 300 and 1,000 μg/kg blunted the effects of fentanyl+alcohol on inspiratory time, peak inspiratory flow, and end-expiratory pause. Naloxone at 300 μg/kg potentiated fentanyl+alcohol effects on expiratory time, relaxation time, and end-inspiratory pause. Naloxone at 1,000 μg/kg potentiated fentanyl+alcohol effects on peak expiratory flow, relaxation time, and end-inspiratory pause.

Altogether, these data indicate that high doses of naloxone are necessary to partially rescue the ventilatory alterations caused by fentanyl+alcohol, but naloxone treatment worsens expiratory-related measures. Effects of 300 μg/kg naloxone on 25 μg/kg fentanyl alone and 0.59 g/kg alcohol alone are shown in Supplemental Figures 5–7.

### Naloxone injected before fentanyl+alcohol did not fully prevent fentanyl+alcohol–induced impairment of minute ventilation or apneic pauses

#### Analysis over time.

**Figure 4:**
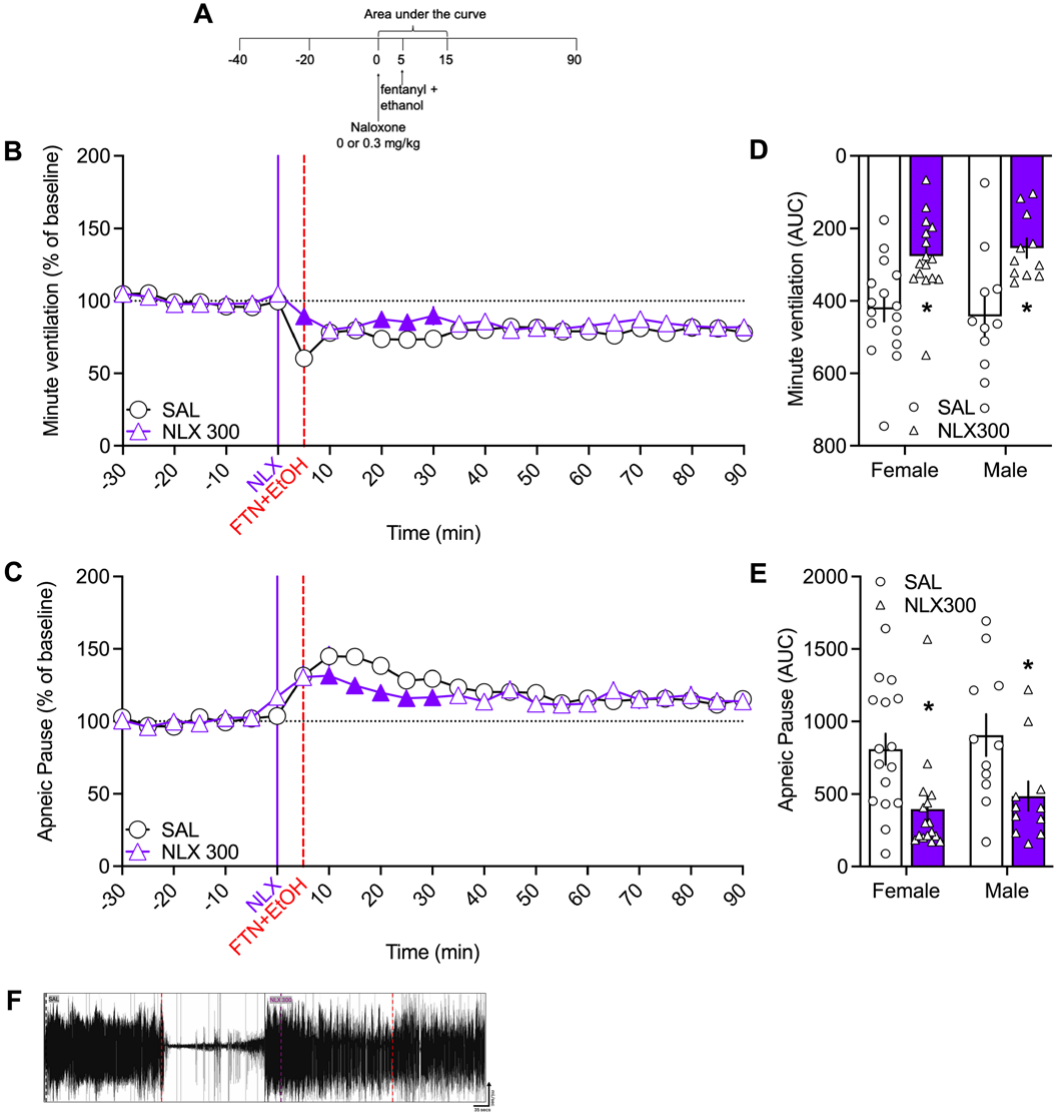
Effect of pretreatment with naloxone on effects of fentanyl+alcohol (25 μg/kg + 0.59 g/kg) on minute ventilation and apneic pauses. Rats received an injection of naloxone (0 and 300 μg/kg) followed by an i.v. infusion of fentanyl+alcohol (25 μg/kg + 0.59 g/kg, 2.5 mL/kg). (**A**) Timeline of each test. (**B**) Pretreatment with 300 μg/kg naloxone attenuated the fentanyl+alcohol–induced decrease in minute ventilation in a time-dependent manner. (**C**) Pretreatment with 300 μg/kg naloxone attenuated the fentanyl+alcohol–induced increase in apneic pauses in a time-dependent manner. The data are expressed as the mean ± SEM and were analyzed by 2-way RM-ANOVA followed by Duncan’s post hoc test when appropriate. Filled symbols are different from vehicle (*P* < 0.05). (**D**) AUC of the first 15 min after fentanyl+alcohol infusion for minute ventilation. (**E**) AUC of the first 15 min after fentanyl+alcohol infusion for apneic pauses. The data are expressed as the mean ± SEM and were analyzed by 2-way RM-ANOVA. Main treatment effect: *P < 0.05, naloxone vs. saline. *n* = 16 females, 12 males. (**F**) Representative raw plethysmography traces.

For minute ventilation, the 2-way RM-ANOVA revealed no main effect of time or of sex, no sex × group interaction, no time × sex interaction, and no sex × group × time interaction. However, there was a main effect of naloxone on fentanyl+alcohol–induced minute ventilation (*F*_1,25_ = 6.642, *P* = 0.0162), and there was a treatment × time interaction (*F*_17,425_ = 2.813, *P* = 0.0002). Naloxone increased minute ventilation at 0 and 15–25 min compared with saline (Figure 4B).

For apneic pauses, the 2-way RM-ANOVA revealed a main effect of time on fentanyl+alcohol–induced pauses (*F*_17,435_ = 9.228, *P* < 0.0001). There was no main effect of naloxone on fentanyl+alcohol–induced apnea compared with saline (*F*_1,25_ = 1.148, *P* = 0.294). There was a treatment × time interaction (*F*_17,425_ = 2.472, *P* = 0.001). Naloxone decreased apnea at 5–25 min (Figure 4C). There was no effect of sex, no sex × group interaction, no time × sex interaction, and no sex × group × time interaction.

#### AUC.

For minute ventilation, the AUC analyses revealed no main effect of sex and no sex × treatment interaction. There was a main effect of naloxone in attenuating the fentanyl+alcohol–induced impairment of minute ventilation (*F*_1,25_ = 32.48, *P* < 0.0001; Figure 4D). For apneic pause, the AUC analysis revealed no effect of sex and no sex × treatment interaction (Figure 4E) but showed a main effect of naloxone (*F*_1,25_ = 15.41, *P* = 0.0006).

The AUCs for the other 9 parameters are shown in Table 5. Pretreatment with naloxone blunted the effects of fentanyl+alcohol (decreased the AUC) on frequency, inspiratory time, peak inspiratory flow, and end-expiratory pause and potentiated its effects on end-inspiratory pause. Altogether, these data indicate that naloxone is partially effective in preventing the ventilatory alterations caused by fentanyl+alcohol.

### Effect of 25 μg/kg fentanyl in fentanyl- and alcohol-dependent rats

Next, we tested the effects of fentanyl, alcohol, and their combination in fentanyl- and alcohol-dependent groups compared with a nondependent group. Alcohol and fentanyl dependence had no effect on baseline ventilatory parameters (Supplemental Figure 8).

#### Analysis over time.

**Figure 5:**
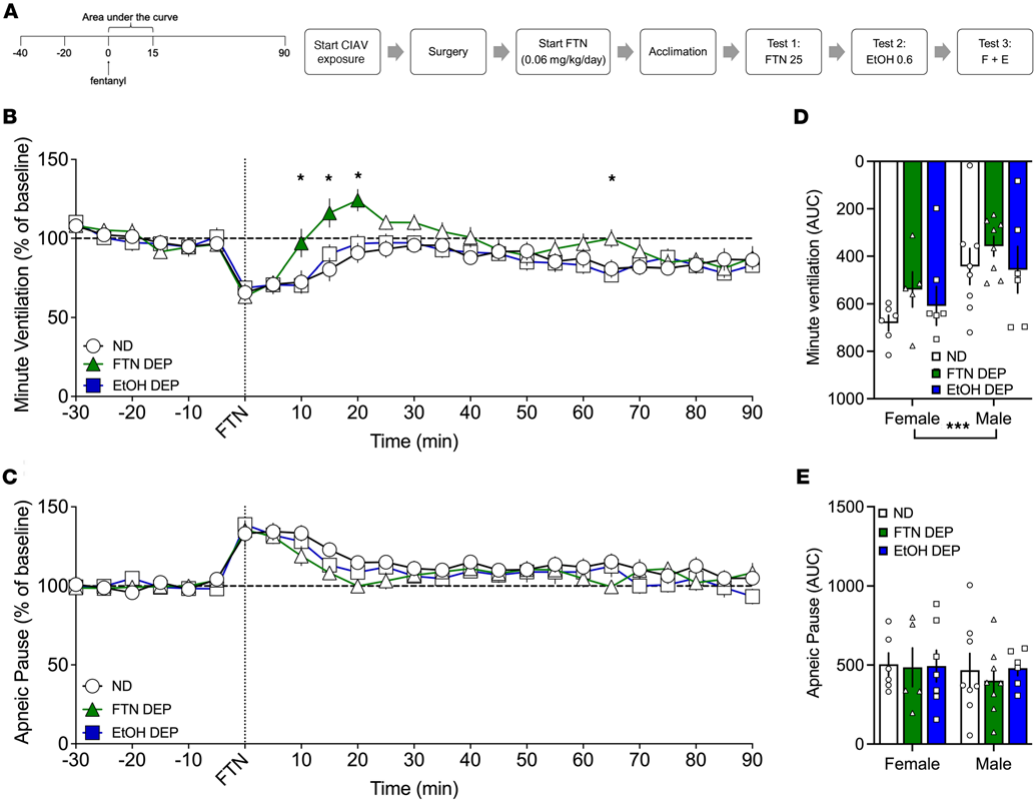
Effect of 25 μg/kg fentanyl in fentanyl- and alcohol-dependent and nondependent rats. Rats received an i.v. infusion of fentanyl (25 μg/kg, 2.5 mL/kg). (**A**) Timeline of each test. CIAV, chronic, intermittent alcohol vapor. (**B**) Fentanyl decreased minute ventilation in a time-dependent manner. (**C**) Fentanyl increased apneic pauses in a time-dependent manner. The data are expressed as the mean ± SEM and were analyzed by 2-way RM-ANOVA followed by Duncan’s post hoc test when appropriate. Filled symbols are different from nondependent (*P* < 0.05). *P < 0.05, fentanyl-dependent vs. alcohol-dependent. (**D**) AUC of the first 15 min after infusion for minute ventilation. (**E**) AUC of the first 15 min after infusion for apneic pauses. The data are expressed as the mean ± SEM and were analyzed by 2-way RM-ANOVA followed by Šidák’s post hoc test when appropriate. *n* = 5–7 females, 5–7 males.

For minute ventilation, the RM-ANOVA showed a main effect of time (*F*_18,684_ = 13.231, *P* < 0.0001) but no main effect of group (*F*_2,38_ = 3.063, *P* = 0.0568). There was a group × time interaction (*F*_36,684_ = 2.0408, *P* = 0.0004; Figure 5B). The fentanyl-dependent group exhibited a faster recovery of minute ventilation 10–20 min after infusion compared with the nondependent group (*P* < 0.05). For apneic pauses, the RM-ANOVA showed a main effect of time (*F*_18,666_ = 15.417, *P* < 0.0001), no main effect of group (*F*_2,37_ = 1.023, *P* = 0.369), and no group × time interaction effect (*F*_36,666_ = 1.065, *P* = 0.369; Figure 5C).

#### AUC.

For minute ventilation, the AUC analyses for the first 15 min after fentanyl infusion showed a main effect of sex (*F*_1,35_ = 11.22, *P* = 0.002) with females showing a greater decrease than males. There was no effect of group (*F*_2,35_ = 1.409, *P* = 0.26) and no sex × group interaction (*F*_2,35_ = 0.207, *P* = 0.81; Figure 5D). For apneic pauses, the area under analyses for the first 15 min after fentanyl infusion showed no effect of group or sex and no sex × group interaction (Figure 5E).

The AUCs for the other 9 parameters are shown in Table 6. Compared with the nondependent group, chronic exposure to alcohol and fentanyl led to lower AUC for (i.e., reduced effect on) frequency of breathing, expiratory time, and relaxation time in response to bolus fentanyl injection.

### Effect of 0.59 g/kg alcohol in fentanyl- and alcohol-dependent rats

#### Analysis over time.

**Figure 6:**
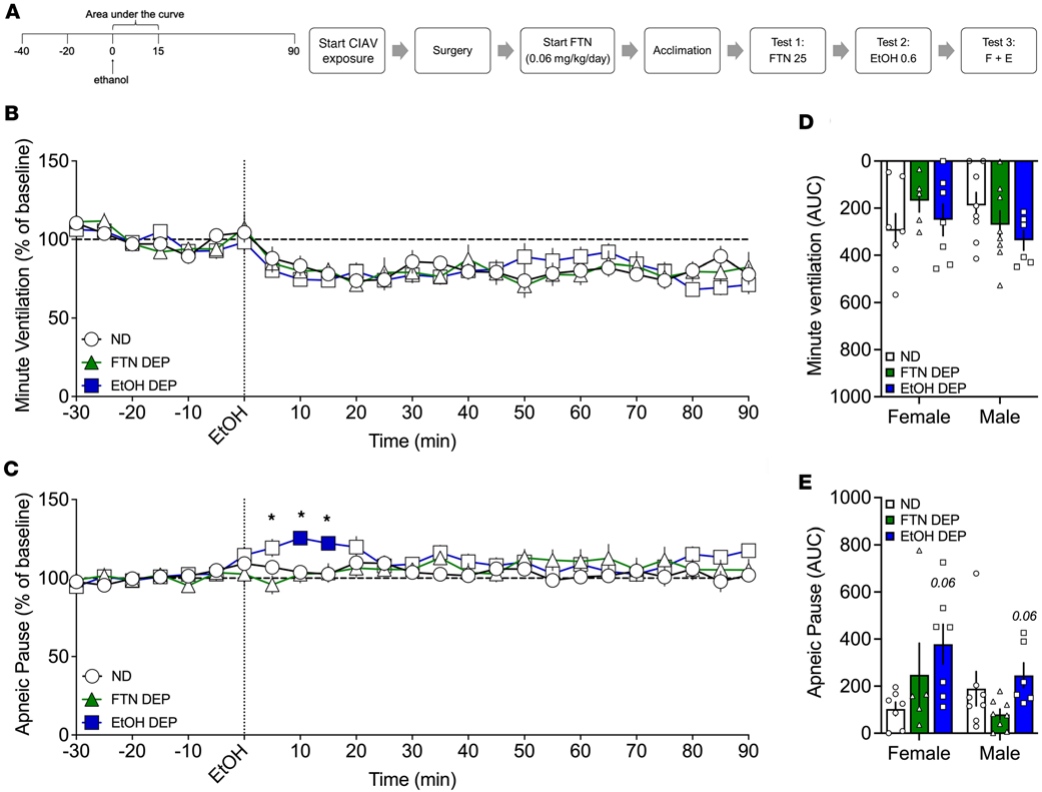
Effect of 0.59 g/kg alcohol in fentanyl- and alcohol-dependent and nondependent rats. Rats received an i.v. infusion of alcohol (0.59 g/kg, 2.5 mL/kg). (**A**) Timeline of each test. CIAV, chronic, intermittent alcohol vapor. (**B**) Alcohol did not affect minute ventilation. (**C**) Alcohol increased apneic pauses in a time-dependent manner. The data are expressed as the mean ± SEM and were analyzed by 2-way RM-ANOVA followed by Duncan’s post hoc test when appropriate. Filled symbols are different from nondependent (*P* < 0.05). *P < 0.05, fentanyl-dependent vs. alcohol-dependent. (**D**) AUC of the first 15 min after infusion for minute ventilation. (**E**) AUC of the first 15 min after infusion for apneic pauses. The data are expressed as the mean ± SEM and were analyzed by 2-way RM-ANOVA followed by Šidák’s post hoc test when appropriate. *n* = 5–7 females, 5–8 males.

The 2-way RM-ANOVA showed a main effect of time on minute ventilation (*F*_18,684_ = 13.231, *P* < 0.0001) but no main effect of group (*F*_2,38_ = 0.023, *P* = 0.977). There was no group × time interaction (*F*_36,684_ = 1.092, *P* = 0.329; Figure 6B). The 2-way RM-ANOVA of the effects of alcohol over time on apneic pauses showed no main effect of time (*F*_18,684_ = 0.824, *P* = 0.673) or main effect of group (*F*_2,38_ = 2.253, *P* = 0.119). However, there was a significant group × time interaction in response to alcohol (*F*_36,684_ *=* 1.610, *P* = 0.014; Figure 6C). Duncan’s post hoc test showed that the alcohol-dependent group exhibited an increase in apneic pauses at 10 and 15 min after infusion (*P* < 0.05) compared with the nondependent group.

#### AUC.

For minute ventilation, the AUC for the first 15 min after alcohol infusion showed no main effect of group (*F*_2,35_ = 0.722, *P* = 0.49), no main effect of sex (*F*_1,35_ = 0.299, *P* = 0.59), and no sex × group interaction (*F*_2,35_ = 1.914, *P* = 0.16; Figure 6D). For apneic pauses, the AUC for the first 15 min after alcohol infusion showed a significant main effect of group (*F*_2,35_ = 3.486, *P* = 0.042; Figure 6E). Šidák’s post hoc test showed that the alcohol-dependent group exhibited a trend toward more severe apnea than the nondependent group (*P* = 0.057). There was no main effect of sex (*F*_1,35_ = 1.618, *P* = 0.21) and no sex × group interaction (*F*_2,35_ = 2.42, *P* = 0.104).

The AUCs for the other 9 parameters are shown in Table 7. Compared with the nondependent group, chronic exposure to alcohol led to higher AUC for (i.e., sensitization effect on) end-expiratory pause.

### Effect of fentanyl+alcohol (25 μg/kg + 0.59 g/kg) in fentanyl- and alcohol-dependent rats

#### Analysis over time.

**Figure 7:**
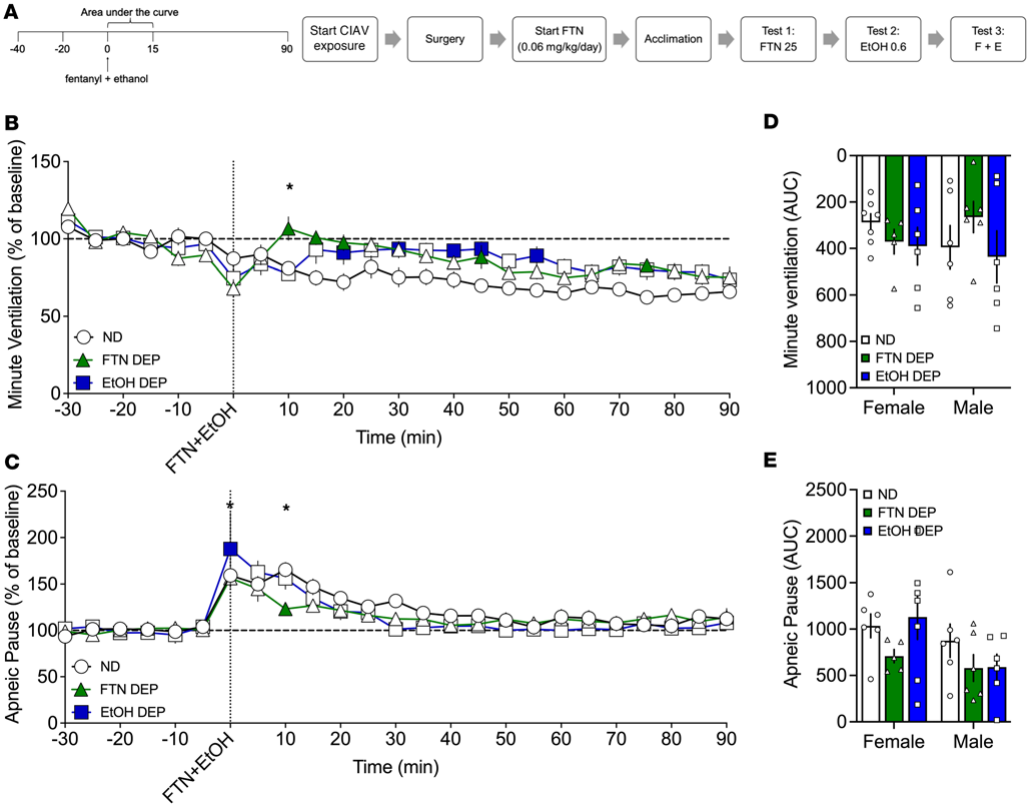
Effect of fentanyl+alcohol (25 μg/kg + 0.59 g/kg) in fentanyl-dependent, alcohol-dependent, and nondependent rats. Rats received an i.v. infusion of a fentanyl+alcohol combination (25 μg/kg and 0.59 g/kg, respectively, 2.5 mL/kg). (**A**) Timeline of each test. CIAV, chronic, intermittent alcohol vapor. (**B**) Fentanyl+alcohol decreased minute ventilation in a time-dependent manner. (**C**) Fentanyl+alcohol increased apneic pauses in a time-dependent manner. The data are expressed as the mean ± SEM and were analyzed by 2-way RM-ANOVA followed by Duncan’s post hoc test when appropriate. Filled symbols are different from nondependent (*P* < 0.05). *P < 0.05, fentanyl-dependent vs. alcohol-dependent. (**D**) AUC of the first 15 min after infusion for minute ventilation. (**E**) AUC of the first 15 min after infusion for apneic pauses. The data are expressed as the mean ± SEM and were analyzed by 2-way RM-ANOVA followed by Šidák’s post hoc test. *n* = 5–7 females, 5–7 males.

For minute ventilation, the RM-ANOVA showed a main effect of time (*F*_18,576_ = 6.244, *P* < 0.0001), a main effect of group (*F*_2,32_ = 7.693, *P* = 0.002), and a significant group × time interaction (*F*_36,544_ = 2.361, *P* < 0.0001). Duncan’s post hoc test showed that the fentanyl-dependent group had higher minute ventilation at 0, 10, 15, 20, 45, and 75 min after infusion compared with the nondependent group. In contrast, the alcohol-dependent group exhibited higher minute ventilation at 20, 30, 40, 45, and 55 min after infusion (Figure 7B). For apneic pauses, the RM-ANOVA showed a main effect of time (*F*_18,522_ = 19.025, *P* < 0.0001), no main effect of group (*F*_2,29_ = 0.711, *P* = 0.499), and a significant group × time interaction (*F*_36,522_ *=* 1.557, *P* = 0.022; Figure 7C). Duncan’s post hoc test showed that the alcohol-dependent group exhibited an increase in apneic pauses at 0 min after infusion compared with the nondependent group (*P* < 0.05), whereas the fentanyl-dependent group exhibited a decrease in apneic pauses at 10 min after infusion.

#### AUC.

For minute ventilation, the AUC for the first 15 min after fentanyl+alcohol infusion showed no main effect of group (*F*_2,30_ = 0.800, *P* = 0.46) or sex (*F*_1,30_ = 0.061, *P* = 0.81) and no sex × group interaction (*F*_2,30_ = 0.963, *P* = 0.39; Figure 7D). For apneic pause, the AUC for the first 15 min after fentanyl+alcohol infusion showed no effect of group (*F*_2,30_ = 1.642, *P* = 0.21; Figure 7E), no effect of sex (*F*_1,30_ = 3.894, *P* = 0.058), and no sex × group interaction (*F*_2,30_ = 1.642, *P* = 0.21).

The AUCs for the other 9 parameters are shown in Table 8. Compared with the nondependent group, chronic exposure to fentanyl had a reduced effect on expiratory time and end-expiratory pause.

### Blood gasometry and blood and brain drug concentrations following fentanyl, alcohol, and fentanyl+alcohol

**Figure 8:**
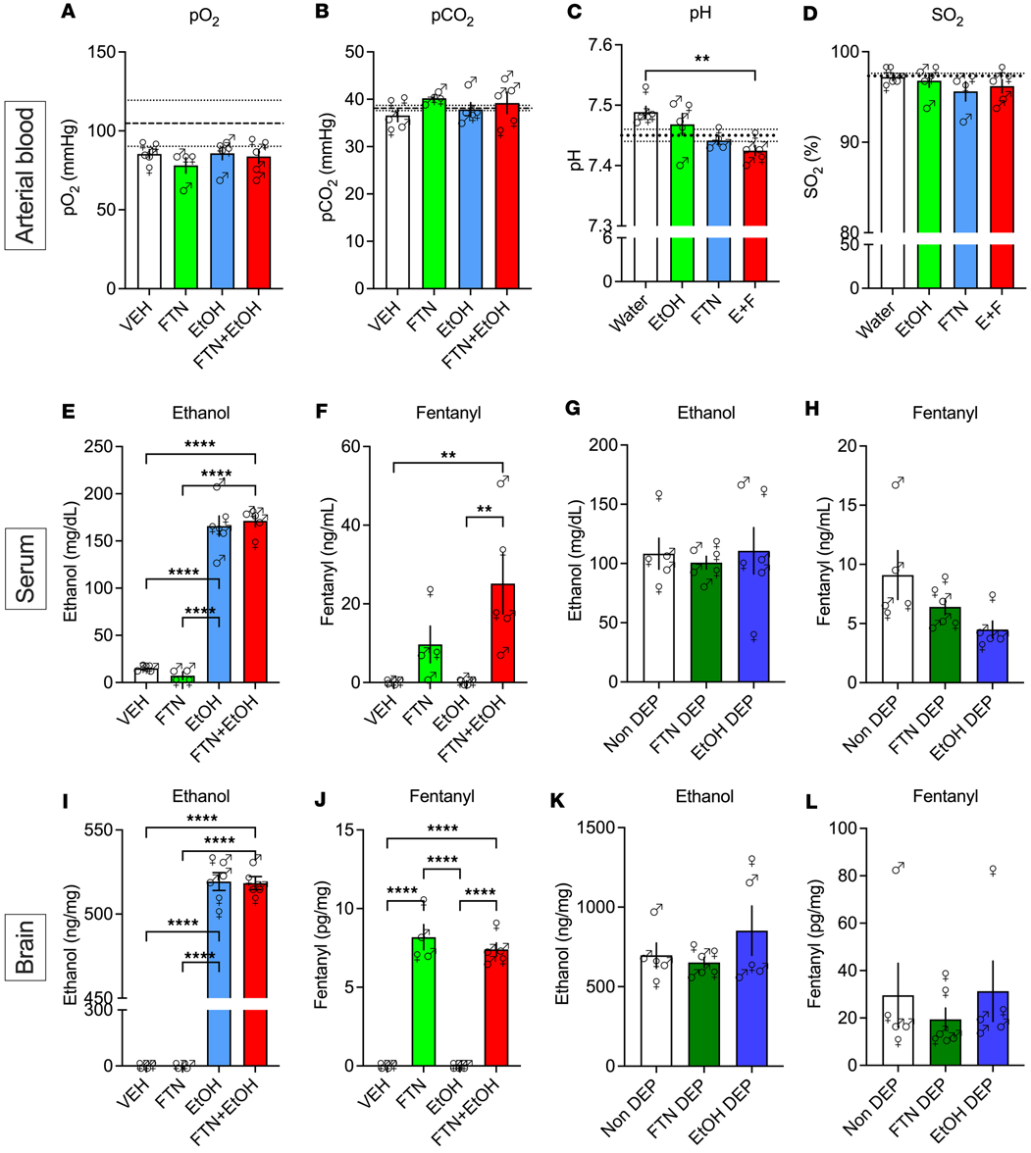
Effects of 25 μg/kg fentanyl and 0.59 g/kg alcohol on arterial partial pressure gasometry and serum and brain fentanyl and alcohol levels. Rats received i.v. infusions of sterile water (2.5 mL/kg), fentanyl (25 μg/kg, 2.5 mL/kg), alcohol (30% vol/vol, 0.59 g/kg, 2.5 mL/kg), and fentanyl+alcohol (25 μg/kg + 0.59 g/kg, 2.5 mL/kg). Arterial blood was collected 5 min after the infusion, and trunk blood and brains were collected 10 min after infusion. (**A**–**L**) Effect of water, fentanyl, alcohol, and fentanyl+alcohol on partial pressure of oxygen (pO_2_) (**A**), partial pressure of carbon dioxide (pCO_2_) (**B**), hydrogen ion concentration (pH) (**C**), oxygen saturation (SO_2_) (**D**), alcohol concentration in blood (**E**), fentanyl concentration in blood (**F**), alcohol concentration in blood in nondependent and dependent rats (**G**), fentanyl concentration in blood nondependent and dependent rats (**H**), alcohol concentration in brain (**I**), fentanyl concentration in brain (**J**), alcohol concentration in brain in nondependent and dependent rats (**K**), and fentanyl concentration in brain in nondependent and dependent rats (**L**). The data are expressed as the mean ± SEM and were analyzed by 1-way ANOVA followed by Šidák’s post hoc test. **P < 0.01, ****P < 0.0001. *n* = 10 males, 9 females.

Acute treatment with fentanyl, alcohol, and fentanyl+alcohol did not change arterial blood oxygen pressure (*F*_3,15_ = 0.6788, *P* = 0.5785; Figure 8A), carbon dioxide pressure (*F*_3,15_ = 0.9435, *P* = 0.4443; Figure 8B), or oxygen saturation (*F*_3,15_ = 0.8418, *P* = 0.492; Figure 8D). Fentanyl+alcohol decreased arterial blood pH (*F*_3,15_ = 5.825, *P* = 0.0076; Figure 8C) compared with vehicle (*P* < 0.01).

Acute treatment with alcohol and fentanyl+alcohol led to detectable serum alcohol levels (*F*_3,16_ = 145.4, *P* < 0.0001; Figure 8E). Rats that received alcohol and fentanyl+alcohol had measurable brain alcohol levels (*F*_3,16_ = 6168, *P* < 0.0001; Figure 8I). Rats that received fentanyl and fentanyl+alcohol had measurable blood (*F*_3,15_ = 7.250, *P* = 0.0031; Figure 8F) and brain fentanyl levels (*F*_3,16_ = 142.1, *P* < 0.0001; Figure 8J).

Acute treatment with fentanyl+alcohol led to similar levels of alcohol in blood (*F*_2,14_ = 0.1442, *P* = 0.867; Figure 8G) and brain (*F*_2,13_ = 1.202, *P* = 0.332; Figure 8K) in dependent and nondependent rats and similar fentanyl levels in blood (*F*_2,13_ = 3.186, *P* = 0.0748; Figure 8H) and brain (*F*_2,13_ = 0.3941, *P* = 0.682; Figure 8L) in dependent and nondependent rats.

## Discussion

The present study characterized physiological parameters of respiratory depression in rats caused by the concomitant administration of fentanyl+alcohol compared with fentanyl and alcohol alone in dependent and nondependent rats and examined naloxone’s effectiveness in reversing the hypoventilation caused by each condition. Using doses within the range used by humans and reported in overdoses, the results indicate that naloxone alone may be insufficient to completely reverse overdoses from fentanyl+alcohol combinations.

We found that alcohol potentiated fentanyl-induced respiratory depression (Figure 1) and naloxone did not fully reverse this effect. Notably, the combination of fentanyl and a high (sedative-like) alcohol dose (1.18 g/kg) resulted in greater than 40% mortality, an effect that was not observed when either drug was administered alone, and respiratory depression from this combination was not reversed by naloxone. Fentanyl plus a high alcohol dose produced ventilatory depression in the surviving animals (30% reduction of minute volume and 30% increase in apneic pauses) (Figure 1), and fentanyl+alcohol increased apneic pauses to a greater extent than either drug alone (Figure 1D). However, the rats that died showed a dramatically greater effect, with a 70% reduction in minute volume, mainly driven by a drop in tidal volume, and a 60% increase in apneic pauses (Supplemental Figure 2), suggesting a transient severe hypoventilation or apnea that could have played a critical role in their deaths. In addition, the combined sedative effects of fentanyl+alcohol may further decrease arousal and impair airway patency, consistent with the prolonged loss of righting reflex (Supplemental Figure 1). Furthermore, even a lower (binge-like) alcohol dose (0.59 g/kg) amplified respiratory depression when fentanyl was present. Fentanyl, alcohol, and fentanyl+alcohol reduced minute ventilation and increased apneic pauses (Figure 2).

In the present study, the alcohol-dependent group exhibited a potentiation of apneic pauses. These results are consistent with prior studies in rodents. For example, the combination of alcohol and buprenorphine resulted in marked sedation and respiratory depression that were prevented but not reversed by naloxone (14). In addition, intragastric alcohol administration induced a slow tonic increase in brain oxygen at baseline but strongly potentiated intravenous-heroin-induced oxygen reductions, increasing both the magnitude and the duration of the decrease in oxygen (15).

In the present study, the fentanyl-dependent group exhibited partial tolerance to 25 μg/kg fentanyl and 25 μg/kg fentanyl + 0.59 g/kg alcohol, characterized by a shorter lasting reduction of minute ventilation (20 vs. 10 min), which is consistent with our previous findings (16). In contrast, alcohol dependence potentiated apneic pauses (25% higher in the alcohol-dependent group). This complex interaction between chronic treatment with alcohol and opioids has been demonstrated in mice. Previous studies in male mice have shown that an alcohol liquid diet did not alter respiration alone or the response to the first morphine injection but blocked tolerance to morphine (17). Alcohol alone did not alter ventilation but reversed the tolerance to morphine in mice that were chronically treated with oxycodone (18, 19). Additionally, in female rats, the administration of a high dose of alcohol before repetitive hypoxia exposure altered baseline ventilation, impaired the adaptive ventilatory response to hypoxic exposure, and abolished the long-term increase in ventilatory output that follows intermittent hypoxia exposure (20).

Thus, consistent with the observations in preclinical literature, our data suggest that alcohol dependence sensitizes animals to the respiratory-depressant effects of subsequent alcohol or fentanyl+alcohol exposure. This is likely due to chronic alcohol exposure inducing region-specific changes in GABA-A receptor subunit expression and function, further altering inhibitory control in key brainstem and limbic centers (21). Such adaptations may result in potentially greater respiratory depression similar to the more severe withdrawal symptoms observed, including heightened seizure susceptibility, where repeated cycles of alcohol intoxication and withdrawal sensitize animals to the effects of subsequent exposures (22).

The potentiation of fentanyl’s effects by alcohol in the present study were not explained by changes in pharmacokinetics (Figure 8). Although we found no differences in blood or brain alcohol and fentanyl levels, there are reports in humans of bidirectional pharmacokinetic interactions between opioids and alcohol. Alcohol inhibited heroin metabolism (23, 24), and the absorption of alcohol was decreased by oxycodone (25), but morphine+alcohol coadministration showed no pharmacokinetic interaction (26).

A few human laboratory studies have reported results that are consistent with our preclinical work. In a human laboratory study, morphine administration increased end tidal CO_2_, whereas the effect of alcohol coadministration was inconsistent (26). Setnik et al., in 2014, showed that morphine groups and combined morphine+alcohol groups demonstrated a non-dose-dependent increase in end tidal CO_2_ compared with alcohol alone, and alcohol alone slightly reduced oxygen saturation without changing ventilation. However, in a study of young and elderly participants, oxycodone alone significantly reduced ventilation, whereas oxycodone+alcohol produced a more profound depression of ventilation, hypercapnic response, and oxygen saturation (27).

In humans, opioid-induced respiratory depression is explained as the product of a reduction of respiratory drive, combined with lower levels of consciousness and obstructive sleep apnea (11). Ventilation is controlled by peripheral and central signaling, with multiple sites involved in this control that express MORs and independently exert depressive effects on breathing. The generator of inspiratory rhythm is the pre-Bötzinger complex in the ventrolateral medulla. The integration of peripheral sensory feedback occurs elsewhere in the brainstem, in the nucleus of the solitary tract (28). Alcohol-induced respiratory depression (5, 7) is hypothesized to be mediated by disruptions of the integration of peripheral sensory inputs, such as the hypoxic ventilatory response (13) and hypercapnic drive (12).

Apnea, or the cessation of airflow, can be caused by the suppression of central respiratory drive (i.e., central apnea) in the Kölliker-Fuse/parabrachial complex through the activation of MORs (29) or by the collapse of upper airways (i.e., obstructive apnea). The latter can be caused by a loss of tone of the primary upper airway dilator, the genioglossus (tongue) muscle (30). In male rats, alcohol can suppress genioglossus and postural motor muscle activity on state-dependent aspects of CNS function, independent of direct effects on the respiratory network (31). Alcohol’s effects in respiratory accessory muscles could also contribute to apnea.

In humans, opioid+alcohol combinations also exert physiological effects that presumably interact to worsen respiratory depression. Binge-like doses of alcohol decrease oxygen saturation in post-hypoxia challenge, possibly indicating a lower affinity of hemoglobin for oxygen (32). Additionally, both opioids and alcohol cause hypothermia (33), and a high dose of alcohol alone or in combination with oxycodone decreased blood pressure, but only alcohol increased heart rate (25). Therefore, other physiological factors, such as altered thermoregulation, vasodilation, or metabolic suppression, may have further compromised cardiovascular stability and oxygen delivery (34) and could have also contributed to mortality. Together, these results indicate that mortality following fentanyl+alcohol co-exposure likely arises from the synergistic disruption of multiple homeostatic systems, rather than respiratory depression alone.

In the present study, pretreatment with naloxone mitigated some effects of the fentanyl+alcohol combination but did not restore normal respiration. Naloxone transiently reversed the depression of minute ventilation caused by 25 μg/kg fentanyl + 0.59 g/kg alcohol but did not affect apneic pauses. We propose that compensatory increases in tidal volume and inspiratory drive, likely mediated by peripheral chemoreceptor activation, restore minute ventilation despite persistent alteration in expiratory parameters leading to extended post-expiratory pauses.

In humans, oxycodone+alcohol coadministration also leads to more apneic events, particularly in older individuals (27). The increase in apneic pauses caused by fentanyl+alcohol was particularly resistant to reversal and even prevention by naloxone (27), suggesting that naloxone is ineffective in counteracting upper airway collapse. This is consistent with our observation that apneic pauses are resistant to reversal with naloxone.

In a study in male rats, fentanyl induced immediate apnea followed by respiratory depression (35). The apnea was characterized by central expiratory apnea, laryngeal closure, and pharyngeal constriction/collapse, accompanied by chest wall rigidity. The apneic response was abolished by the blockade of vagus nerve C-fiber signals and attenuated by the antagonism of vagal afferent MORs (35, 36).

### Clinical implications

Based on our observations that naloxone alone does not sufficiently reverse respiratory depression under poly-drug administration of fentanyl+alcohol, efforts should be made to consider additional pharmacological approaches to combat the changing landscape of opioid overdoses. The primary goal of overdose reversal is to quickly restore breathing to prevent brain injury, prioritizing lifesaving over the risk of withdrawal. Based on the present findings and the literature, several possibilities may address this challenge. Highly potent synthetic opioids may require fast-acting, longer-lasting reversal agents like intranasal higher-dose naloxone or nalmefene, which are effective in settings with a delayed emergency response (3). For example, in a crossover clinical study, intramuscular naloxone administration was more effective than an intranasal formulation in reversing fentanyl-induced respiratory depression in opioid-naive individuals and chronic opioid users. Signs of precipitated withdrawal were seen only in chronic users (37).

The efficacy of drugs approved for the treatment of opioid and/or alcohol use disorders, buprenorphine and naltrexone, could also be investigated in poly-drug overdoses. Recent in vitro evidence suggests a higher efficacy of naltrexone against synthetic opioids and in the presence of alcohol (38, 39). Buprenorphine has been shown to prevent fentanyl-induced respiratory depression (40, 41), potentially through its partial agonism of, and high affinity for, MORs, and perhaps its antagonism of κ-opioid receptors (42).

Risk factors for drug overdose do not differ between sexes (43), but illicit opioid use within the past 12 months was 5.4% higher for men, illicit manufactured fentanyl use was 4.4% higher for men (44), and opioid overdose deaths are more prevalent in men (45). In the present study, we did not find consistent sex differences in ventilatory measures, but female rats exhibited an overall higher trend in nonspecific, more severe respiratory depression than males (Tables 2 and 6). The discrepancy between our findings in rats and the clinical literature may be explained by more opioid misuse in men (46).

### Conclusion

The combination of fentanyl+alcohol led to reductions in minute ventilation and severe apnea that were not fully reversed by naloxone. These findings improve our understanding of the role of opioid+alcohol combinations in overdoses, which is relevant to research on preventing and reversing overdoses from poly-substance use.

## Methods

Detailed methods are presented in Supplemental Methods and Results.

### Sex as a biological variable.

Male and female Long-Evans rats were used in all experiments (Table 9).

### Animals.

Seven-week-old Long-Evans rats (experiments 1, 2, 3, and 4) or 4-week-old Long-Evans rats (experiment 5) were obtained from Charles River (Table 9). Additionally, 9-week-old Long-Evans pre-catheterized rats (experiment 6) were obtained from Envigo.

### Drugs.

Fentanyl citrate was obtained from the National Institute on Drug Abuse Drug Supply Program (Research Triangle Institute, Research Triangle Park, North Carolina, USA) and dispensed by the National Institute on Drug Abuse Intramural Research Program Pharmacy (Baltimore, Maryland, USA). We also used alcohol (190-proof ethyl alcohol; Warner Graham Co.), naloxone hydrochloride (Tocris Bioscience, Bio-Techne), and 0.9% sterile saline (Hospira).

### Intravenous catheter surgery.

Rats were implanted with an indwelling silastic catheter (Dow Corning) in the right jugular vein under general anesthesia (2%–3% isoflurane in O_2_) as previously described (47).

### Plethysmography apparatus.

Ventilation was noninvasively monitored using 4 whole-body plethysmograph chambers (SCIREQ) as previously described (16). The ventilatory parameters listed in Table 10 were generated using IOX 2.10.0.40 software (emka TECHNOLOGIES).

### Fentanyl dependence.

In experiment 5, rats were made dependent on fentanyl by daily, subcutaneous injections of escalating doses of fentanyl as previously described (16).

### Alcohol vapor exposure.

In experiment 5, rats were made alcohol dependent by chronic, intermittent alcohol vapor exposure as previously described (48).

### General procedure for plethysmography experiments.

After intravenous (i.v.) catheter implantation and recovery, the rats were habituated to the plethysmography chambers in sessions in which ventilation was not monitored. During testing, the rats were acclimated to the chambers for 10 min, followed by 30 min of baseline data collection. Each rat then received all treatments in a within-subjects Latin square design, with tests 1 week apart. Ventilation was monitored for 90 min after infusion. If a rat’s catheter lost patency during testing, it was repaired or replaced, and the drug was readministered in a makeup test on the fifth week of testing. Detailed methods are described in Supplemental Methods and Results.

### Experiment 1: 25 μg/kg fentanyl + 1.18 g/kg alcohol (“high, sedative-like” dose).

Each rat received a 1 min i.v. infusion of sterile water (5 mL/kg), fentanyl (25 μg/kg, 5 mL/kg), alcohol (1.18 g/kg, 5 mL/kg), or a fentanyl+alcohol combination (25 μg/kg + 1.18 g/kg, 5 mL/kg; Figure 1A). Representative traces of raw ventilation curves in response to each drug are shown in Figure 1G. Alcohol dose was determined based on pilot experiment (Supplemental Figure 1).

### Experiment 2: 25 μg/kg fentanyl + 0.59 g/kg alcohol (“binge-like” dose).

In this experiment, half of the alcohol dose was used to achieve human binge-like blood alcohol levels (>80 mg/dL; *n* = 9–11 per sex per drug). Each rat received a 1 min i.v. infusion of sterile water (2.5 mL/kg), fentanyl (25 μg/kg, 2.5 mL/kg), alcohol (0.59 g/kg, 2.5 mL/kg), or a fentanyl+alcohol combination (25 μg/kg + 0.59 g/kg, 2.5 mL/kg; Figure 2A). Representative traces of raw ventilation curves in response to each drug are shown in Figure 2E.

### Experiment 3: 3.125 μg/kg fentanyl + 0.59 g/kg alcohol.

This experiment used a dose of fentanyl that does not cause respiratory depression (i.e., a subeffective dose). Each rat received a 1 min i.v. infusion of sterile water (2.5 mL/kg), fentanyl (3.125 μg/kg, 2.5 mL/kg), alcohol (0.59 g/kg, 2.5 mL/kg), or a fentanyl+alcohol combination (3.125 μg/kg + 0.59 g/kg, 2.5 mL/kg). The dose of fentanyl was based on a pilot experiment shown in Supplemental Figure 3.

### Experiment 4: naloxone reversal of respiratory depression induced by fentanyl+alcohol.

To test whether naloxone reverses respiratory depression caused by a fentanyl+alcohol combination, we tested 2 cohorts of rats with different naloxone doses. Each rat received a 1 min i.v. infusion of a fentanyl+alcohol combination (25 μg/kg + 0.59 g/kg, 2.5 mL/kg). In cohort 1, 5 min later, the rats received a bolus injection (1 mL/kg, i.v.) of naloxone (0 and 100 μg/kg). In cohort 2, the rats received a bolus injection of naloxone (0, 300, and 1,000 μg/kg; Figure 3A). Representative traces of raw ventilation curves in response to each drug are shown in Figure 3J.

Lastly, we tested whether 300 μg/kg naloxone prevents effects of a fentanyl+alcohol combination. Each rat received a bolus injection of naloxone (0 or 300 μg/kg, 1 mL/kg, i.v.), followed by a 1 min i.v. infusion of a fentanyl+alcohol combination (25 μg/kg + 0.59 g/kg, 2.5 mL/kg) 5 min later (Figure 4A). Representative traces of raw ventilation curves in response to each drug are shown in Figure 4F.

### Experiment 5: fentanyl+alcohol in fentanyl- and alcohol-dependent rats.

Rats were made dependent on alcohol as described above. Fentanyl-dependent rats were tested 4–6 hours into withdrawal. Nondependent rats were concomitantly tested. Each rat received a 1 min i.v. infusion of fentanyl (25 μg/kg, 2.5 mL/kg; Figure 5A), alcohol (0.59 g/kg, 2.5 mL/kg; Figure 6A), or a fentanyl+alcohol combination (25 μg/kg + 0.59 g/kg, 2.5 mL/kg; Figure 7A) in this order at 1-week intervals between tests.

### Experiment 6: blood gasometry and blood and brain drug concentration measurements.

Twenty rats (10 females, 10 males) with jugular vein and femoral artery catheters were used. The rats received a 1 min i.v. infusion of fentanyl (25 μg/kg, 2.5 mL/kg), alcohol (0.59 g/kg, 2.5 mL/kg), or a fentanyl+alcohol combination (25 μg/kg + 0.59 g/kg, 2.5 mL/kg). Five minutes after the infusion, arterial blood was collected for the measurement of blood gases. The rats were euthanized 10 min after infusion, and trunk blood and brains were collected.

To measure blood gas levels (Table 11), 100 μL of arterial blood was injected into CG8+ cartridges (Abbott Laboratories) and analyzed using an i-STAT 1 analyzer (Abbott Laboratories). Fentanyl and alcohol levels were measured in blood and brain (see Supplemental Methods and Results).

### Statistics.

A custom-made application (rvent_app) was used to import, bin, compile, plot, and export Microsoft Excel data sheets from the TXT files that were generated and calculated using IOX 2.10.0.40 software (emka TECHNOLOGIES). Prism 8 software (GraphPad) was used for figure preparation. Statistica 13 software (TIBCO) was used for statistical analyses. All data were aggregated into 1 min bins for analysis of the first 15 min post-infusion or in 5 min bins for the whole 90 min session analyses. The data are expressed as the mean ± SEM percentage of baseline values.

For statistical purposes, the analyses of treatment over time included the post-injection data but not the baseline data; we used 3-way repeated-measures analysis of variance (RM-ANOVA), with drug (treatment) and time as within-subjects factors and sex as a between-subjects factor. To determine the effect of treatments on the AUC, we used 2-way RM-ANOVA, with sex as a between-subjects factor and drug (treatment) as a within-subjects factor. Dunnett’s, Duncan’s, and Šidák’s post hoc tests were used when appropriate. Values of *P* < 0.05 were considered statistically significant.

### Study approval.

All procedures were performed according to the *Guide for the Care and Use of Laboratory Animals* (National Academies Press, 2011) and were approved by the National Institute on Drug Abuse, Intramural Research Program, Animal Care and Use Committee (protocol 23-INRB-13).

### Data availability.

All data presented in this article are available in the Supporting Data Values file.

## Author contributions

EVF, LEH, NDV, AJH, LFV, GFK, and RCNM conceptualized the experiments. EVF, LEH, ANM, AGF, and JCMV performed the experiments. LAK and SNJ performed the alcohol and fentanyl quantification experiments. EVF, LEH, AGF, and RCNM compiled and analyzed the data. RCNM wrote and edited the manuscript. All authors edited the manuscript. The order of co–first authors was determined alphabetically.

## Funding support

This work is the result of NIH funding, in whole or in part, and is subject to the NIH Public Access Policy. Through acceptance of this federal funding, the NIH has been given a right to make the work publicly available in PubMed Central.

- Intramural Research Program of the NIH (ZIA-DA000602, National Institute on Drug Abuse, Intramural Research Program, Neurobiology of Addiction Section, principal investigator: GFK).
- Intramural Research Program of the NIH (ZIA-DA000644, National Institute on Drug Abuse/National Institute on Alcohol Abuse and Alcoholism, Intramural Research Program, Stress and Addiction Neuroscience Unit, principal investigator: LFV).
- Center on Compulsive Behaviors, NIH, via the NIH Shared Resource Subcommittee (to RCNM).
- Pathway for Independence Award (1K99DA056618-01A1) from the National Institute on Drug Abuse (to RCNM).

## Supplementary Material

Supplemental data

Supporting data values

## Figures and Tables

**Table 9:**
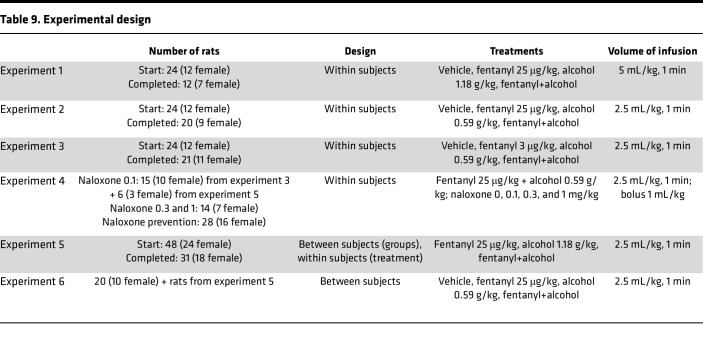
Experimental design

**Table 10:**
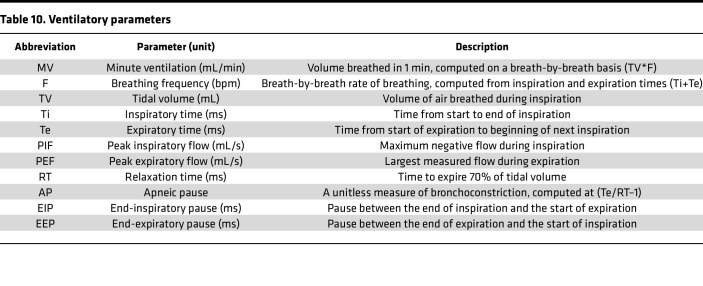
Ventilatory parameters

**Table 11:**
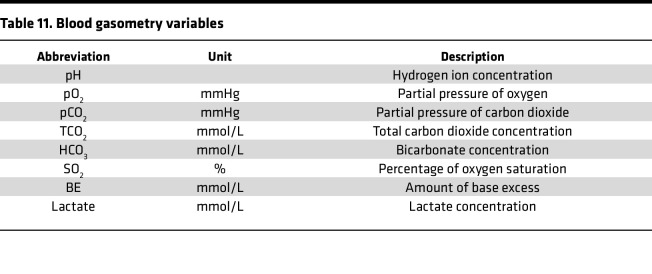
Blood gasometry variables

**Table 1:**
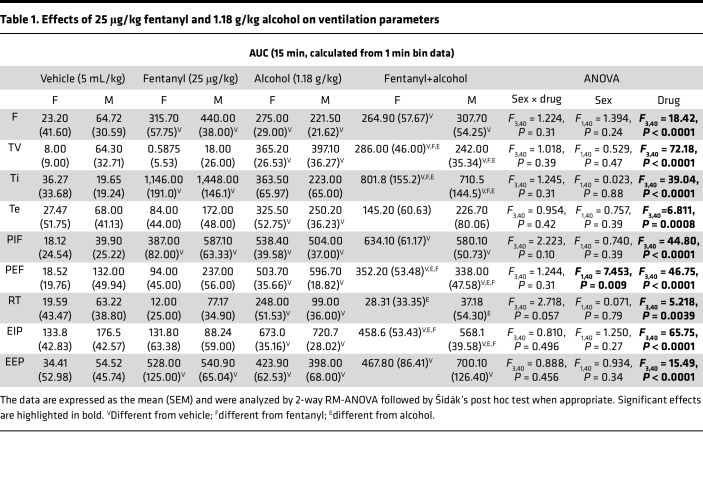
Effects of 25 μg/kg fentanyl and 1.18 g/kg alcohol on ventilation parameters

**Table 2:**
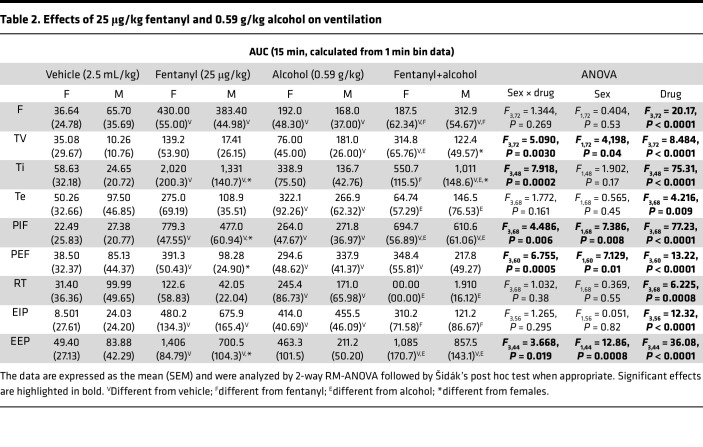
Effects of 25 μg/kg fentanyl and 0.59 g/kg alcohol on ventilation

**Table 3:**
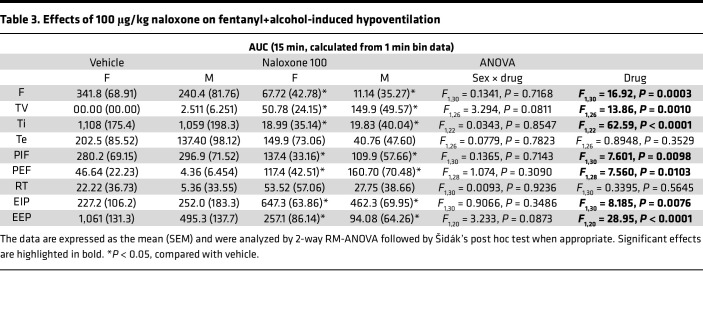
Effects of 100 μg/kg naloxone on fentanyl+alcohol-induced hypoventilation

**Table 4:**
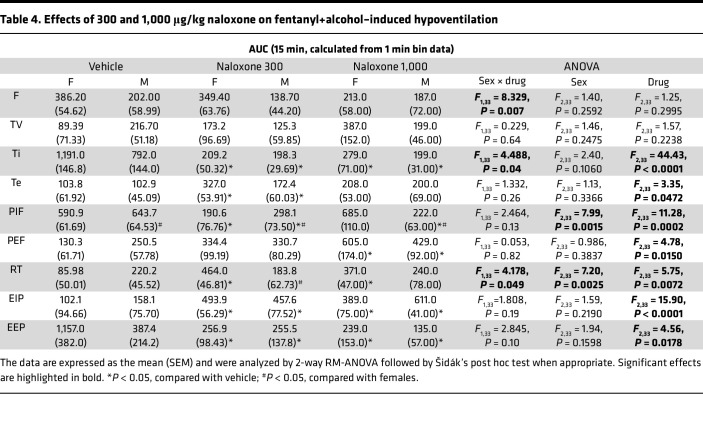
Effects of 300 and 1,000 μg/kg naloxone on fentanyl+alcohol–induced hypoventilation

**Table 5:**
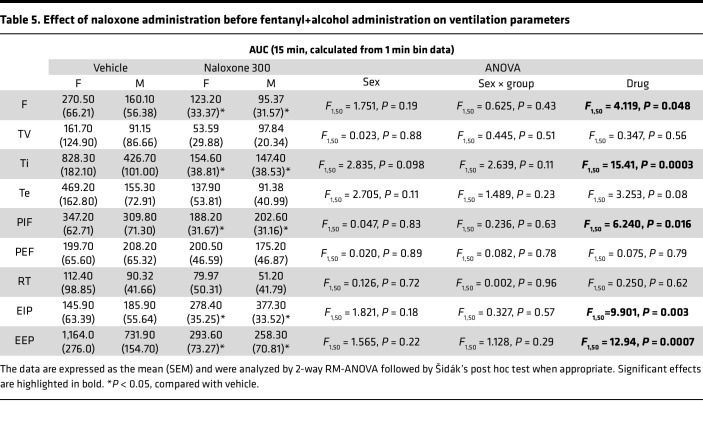
Effect of naloxone administration before fentanyl+alcohol administration on ventilation parameters

**Table 6:**
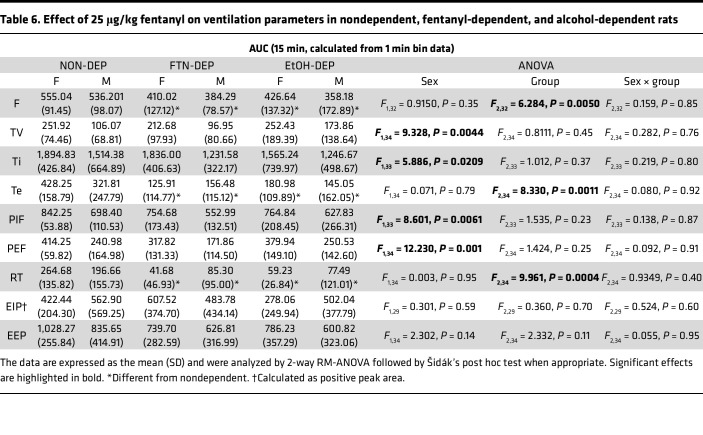
Effect of 25 μg/kg fentanyl on ventilation parameters in nondependent, fentanyl-dependent, and alcohol-dependent rats

**Table 7:**
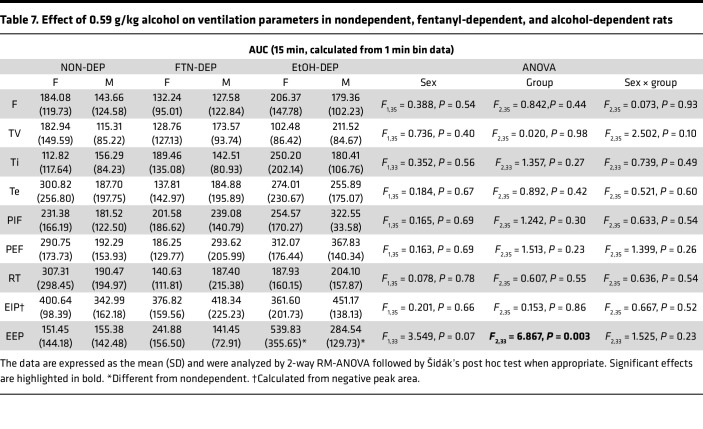
Effect of 0.59 g/kg alcohol on ventilation parameters in nondependent, fentanyl-dependent, and alcohol-dependent rats

**Table 8:**
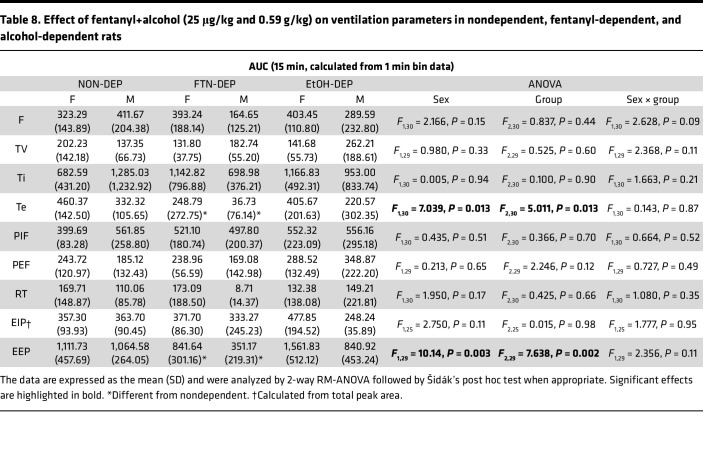
Effect of fentanyl+alcohol (25 μg/kg and 0.59 g/kg) on ventilation parameters in nondependent, fentanyl-dependent, and alcohol-dependent rats

## Version 1 02/03/2026

In-Press Preview

## Version 2 03/23/2026

Electronic publication
