# Supplementary material for: Potentiation of fentanyl-induced respiratory depression by alcohol is not fully reversed by naloxone
Source: JCI Insight. 2026 Feb 3;11(6):e198059. doi: 10.1172/jci.insight.198059 (PMC13043099; doi:10.1172/jci.insight.198059)
Supplement: Supplemental data [file jciinsight-11-198059-s280.pdf]

SUPPLEMENTAL MATERIAL

Potentiation of fentanyl-induced respiratory depression by alcohol is not fully reversed by naloxone

Emma V. Frye,[1, #] Lyndsay E. Hastings,[1, #] Aniah N. Matthews,[1] Adriana Gregory-Flores,[1] Janaina C.M. Vendruscolo,[1] Lindsay A. Kryszak,[2] Shelley N. Jackson,[2] Aidan J. Hampson,[3] Nora D. Volkow,[4] Leandro F. Vendruscolo,[5] Renata C.N. Marchette,[1, *] and George F. Koob[1, *]

[#] Co-first authors *Co-corresponding Authors

[1]Neurobiology of Addiction Section, National Institute on Drug Abuse, Intramural Research Program, National Institutes of Health, Baltimore, MD

[2]Translational Analytical Core, National Institute on Drug Abuse, Intramural Research Program, National Institutes of Health, Baltimore, MD

[3]Division of Pharmacotherapeutic Development, National Institute on Drug Abuse, Rockville, MD

[4]Laboratory for Neuroimaging, National Institute on Alcohol Abuse and Alcoholism, Intramural Research Program, National Institutes of Health, Bethesda, MD

[5]Stress & Addiction Neurobiology Unit, National Institute on Drug Abuse and National Institute on Alcohol Abuse and Alcoholism, Intramural Research Programs, National Institutes of Health, Baltimore, MD

METHODS AND RESULTS

Animals

Seven-week-old Long-Evans male and female rats (Experiments 1, 2, 3, and 4; described below) or 4-week-old Long-Evans male and female rats (Experiment 5; described below) were obtained from Charles River (Kingston, NY, USA; **Table 1**). Additionally, 9-week-old Long-Evans male and female pre-catheterized rats (Experiment 6; described below) were obtained from Envigo (Indianapolis, IN, USA). The rats were group housed (Experiments 1, 2, 3, 4, and 5) or single housed (Experiment 6) at 21°C ± 2°C with free access to food and water, except during the tests. Separate cohorts were used for Experiments 1-4 and 6, but some rats in Experiment 4 had previously been used in Experiment 3 or 5. Rats in Experiments 1, 2, 3, 4, and 6 were kept on a 12 h/12 h light/dark cycle (lights on at 6 AM), and respiratory tests were conducted during the light cycle. Rats in Experiment 5 were kept on a reverse 12 h/12 h light/dark cycle (lights on at 8 PM), and behavioral tests were conducted during the dark cycle. Rats in Experiment 5 were tested on a reverse light/dark cycle.

Drugs

Fentanyl citrate was diluted in 0.9% sterile saline for intravenous (i.v.) injections (1.25-25 µg/kg, 1-5 mL/kg) and subcutaneous (s.c.) administration (0.02-0.06 mg/kg, 1 mL/kg). Fentanyl was obtained from the National Institute on Drug Abuse Drug Supply Program (Research Triangle Institute, Research Triangle Park, NC, USA) and dispensed by the National Institute on Drug Abuse Intramural Research Program Pharmacy. Alcohol (190 proof ethyl alcohol; Warner-Graham Company, Cockeysville, MD, USA) was diluted in sterile water (Hospira, Lake Forest, IL, USA) to a concentration of 30% v/v and administered i.v. at 2.5-5 mL/kg. We used i.v. alcohol administration to achieve precise dosing and timing control, thereby reducing variability in blood alcohol

concentrations. This approach also allowed us to stay consistent with blood alcohol concentrations found in postmortem human polydrug overdoses (1). Additionally, i.v. administration minimized confounds related to gastrointestinal absorption variability that are inherent to oral or gastric routes. The doses of alcohol were selected based on a pilot study (**Supplemental figure 1**) to achieve blood alcohol levels (BALs) consistent with binge-like and sedation-like levels. Naloxone was diluted in 0.9% sterile saline (Hospira, Lake Forest, IL, USA) for i.v. injections (100 μg/mL/kg; Tocris Bioscience, Bio-Techne, Minneapolis, MN, USA).

Intravenous catheter surgery

Rats were implanted with an indwelling silastic catheter (Dow Corning, Midland, MI, USA) in the right jugular vein (2) under general anesthesia (2-3% isoflurane in O_2). The analgesic meloxicam (1 mg/kg, 1 mg/mL, s.c.; Hospira, Lake Forest, IL, USA) was given immediately before surgery. The catheter was anchored to the vein with suture thread and passed subcutaneously to exit dorsally on the back of the rat. Catheters were flushed daily with flushing solution: 0.3 mL of a 0.9% sterile saline solution that contained the anticoagulant heparin (30 USP units/mL; Sagent Pharma, Schaumburg, IL, USA) and the antibiotic gentamicin (4.25 mg/mL; Hospira, Lake Forest, IL, USA). If catheter patency failed during the study, the animal was re-catheterized in the left jugular vein.

Plethysmography apparatus

Ventilation was noninvasively monitored using four whole-body plethysmograph chambers (20 cm diameter ´ 13 cm height, 3 L volume; SCIREQ, Montreal, QC, Canada). Each chamber contained a tower attachment (15 cm) through which 30 cm of Tygon tubing was connected to a liquid swivel and protected by a metal spring. The tubing was connected to the rat's i.v. catheter to permit intravenous drug injection without opening the chamber and to allow the rat to move and turn freely within the

chamber. The plethysmograph detected changes in pressure and airflow within the chamber relative to a reference chamber, and the data were used to generate the ventilatory parameters that are listed in **Table 2** using IOX 2.10.0.40 software (emka TECHNOLOGIES, Sterling, VA, USA). Ventilatory parameters were continuously monitored during 2.5 h sessions. The apneic pause was calculated as an index of post-expiratory breathing cessation, using the formula: Apneic pause = (expiratory time/relaxation time) − 1, relaxation time is defined as the time required for 65% of the expiratory volume to be exhaled. This metric reflects the duration of the pause between the end of expiration and the onset of the subsequent inspiration. Under normal breathing conditions, expiratory time and relaxation are proportional, resulting in apneic pause values close to zero. Increased apneic pause values indicate a lengthened post-expiratory pause and reduced respiratory drive.

Blood alcohol levels:

For the assessment of blood alcohol levels (BALs) following intravenous alcohol administration, we used 4 males and 3 females Long Evans rats that received either two infusions, 5 min apart, of alcohol 20% at a volume of 5 mL/kg resulting in a dose of 1.59 g/kg (2 M, 1 F) or alcohol 30% at a volume of 5 mL/kg resulting in a dose of 1.18 g/kg. Five minutes after the last infusion, the distal end of the tail was clipped and blood collected into centrifuge tubes and serum was transferred to a clean tube. An alcohol calibration curve was prepared from 12.5-300 mg/dl (serum) using alcohol standards (Cerilliant, Round Rock, TX, USA) in water. Briefly, 10 µL of alcohol standard or serum was added to 10 ml glass headspace vials (Agilent, Santa Clara, CA, USA) and sealed with a crimp cap. The vials were heated in a 70°C 7697 A Headspace Sampler (Agilent) before headspace injection onto an MXT-Volatiles 30 m, 0.28 mm inner diameter, 1.25 µm df column (Restek, Center County, PA, USA) using helium as the carrier gas. The 8890 gas-chromatography system column oven (Agilent) was heated to 40°C for an isocratic 6 min run that was paired with a 5977B gas-chromatograph/mass selective

detector (Agilent). Alcohol concentrations were measured in positive ion mode with an electron impact source at 31.1 *m/z* as the quantifier and 45.1 and 46.1 *m/z* as the qualifiers.

The 1.59 g/kg dose of alcohol resulted in a mean BAL of 256 mg/dL, whereas the 1.18 g/kg dose resulted in a mean BAL of 183 mg/dL (**Supplemental Figure 1**).

Loss of righting reflex (LORR):

To evaluate the loss of righting reflex (LORR), 4 male and 4 female Long Evans rats, 6 weeks old at the beginning of protocol, received alcohol 1.18 g/kg (30%, 5 mL/kg, i.v.), fentanyl (12 and 25 ug/kg, 5 mL/kg, i.v.) in the different combinations outlined below. Tests were performed 3 to 4 days apart. Immediately after injection, the animals were placed on their backs in a supine position on a V-shaped plexiglass platform. The amount of time to right themselves to an upright position on all four legs was recorded. Treatment combinations: saline + saline, alcohol + saline, saline + fentanyl 12, saline + fentanyl 25, alcohol + fentanyl 12, and alcohol + fentanyl 25.

Only the alcohol and fentanyl combination led to prolonged LORR. Alcohol 1.18 g/kg combined with fentanyl 12 μg/kg resulted in average LORR of 48.7 sec while alcohol 1.18 g/kg combined with fentanyl 25 μg/kg resulted in average LORR of 318.6 sec (**Supplemental Figure 1**).

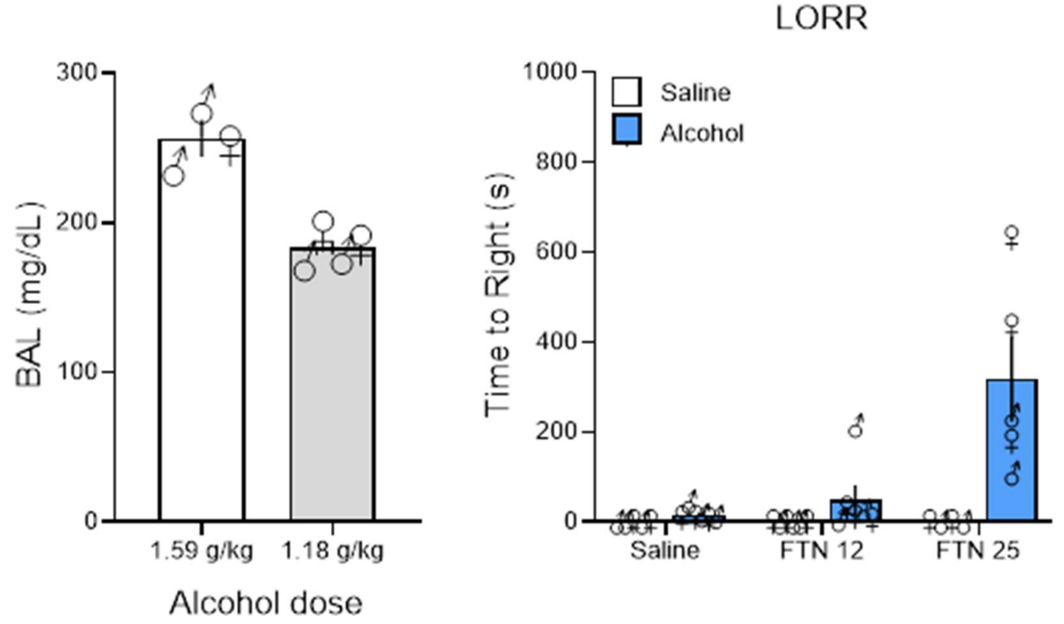

Supplemental Figure 1. Blood alcohol levels and loss of righting reflex (LORR). Blood alcohol levels (BAL) were measured through tail bleeds 5 min after the alcohol infusion. The LORR was measured immediately after injections of saline, fentanyl, alcohol, or fentanyl + alcohol. Cutoff was set at 15 min post infusion. Data are expressed and mean ± SEM.

Fentanyl dependence

In Experiment 5, 8 male and 7 female rats were made dependent on fentanyl by daily, s.c. injections of escalating doses of fentanyl. Injections started 1 week after i.v. catheter surgery. Fentanyl injections at 0.02 and 0.04 mg/kg were administered over the first 2 days, and then the rats were maintained at 0.06 mg/kg, 5 days/week, for the remainder of the study. The dose of fentanyl was based on the amount of fentanyl that was taken in extended-access (12 h) operant self-administration studies, and it was used in our previous study of respiratory depression that was induced by fentanyl and heroin and assessed by whole-body plethysmography (3, 4).

Alcohol vapor exposure

In Experiment 5, 8 male and 7 female rats were made alcohol-dependent by chronic, intermittent alcohol vapor exposure as previously described (5–7). The rats were exposed to daily cycles of 14 h of alcohol vapor, followed by 10 h of room air, for 2 weeks before i.v. catheter surgery. Blood alcohol levels ranged between 100 and 300 mg/dl throughout vapor exposure. Testing occurred once weekly, 4-6 h into alcohol withdrawal, a timepoint at which brain and blood alcohol levels have been reported to be negligible (8). Nondependent and fentanyl-dependent rats in Experiment 5 were not exposed to alcohol vapor but underwent behavioral testing at approximately the same time as the alcohol-dependent group. This vapor model of alcohol dependence produces both somatic and affective symptoms of alcohol dependence (9, 10).

Experiment 1: 25 μg/kg fentanyl + 1.18 g/kg alcohol (5 mL/kg, "high, sedative-like, dose")

Each rat received a 1 min i.v. infusion of sterile water (5 mL/kg), fentanyl (25 μg/kg, 5 mL/kg), alcohol (1.18 g/kg, 5 mL/kg), or a combination of fentanyl and alcohol (25 μg/kg + 1.18 g/kg, 5 mL/kg) in a within-subjects Latin-square design. This dose of alcohol was selected to produce BALs of 180-200 mg/dL. Ventilation was monitored for 90 min post-administration. The same procedure was repeated for a total of 4 consecutive weeks, with a single drug exposure per week, until all the rats received every drug (*n* = 5-7/sex/drug). The fentanyl dose was chosen based on our previous study (3), and the dose of alcohol for Experiment 1 was chosen to reach a blood alcohol level of approximately 200 mg/dL (**Supplemental Figure 1**). The rats weighed 224.2 ± 19.5 g (mean ± SD) and 192.3 ± 10.0 g (male and female, respectively) at the start of the study. By the end of the study, males gained an average of 171.8 g, and females gained an average of 83.8 g, and the mortality rate was 41.7% for females and 44.4% for males (**Figure 1B**).

The combination of 25 μg/kg fentanyl and the high dose of 1.18 g/kg alcohol resulted in significant mortality (41.7% females, 33.3% males; **Figure 1B).** Alcohol (1.18 g/kg) alone and 25 μg/kg fentanyl alone produced no mortality. **Supplemental Figure 2A** shows data of the first 15 min following infusion from rats that did not complete all tests. **Supplemental Figure 2B** shows the response to 25 μg/kg fentanyl + 1.18 g/kg alcohol in the first 2 min following infusion in rats that died and the ones that survived. **Supplemental Figure 2C** shows the time-dependent analysis of minute ventilation following water, 25 μg/kg fentanyl + 1.18 g/kg alcohol in the surviving rats and 25 μg/kg fentanyl + 1.18 g/kg alcohol in the deceased rats. **Supplemental Figure 2D** shows the time-dependent analysis of apneic pause following water, 25 μg/kg fentanyl + 1.18 g/kg alcohol in the surviving rats and 25 μg/kg fentanyl + 1.18 g/kg alcohol in the deceased rats.

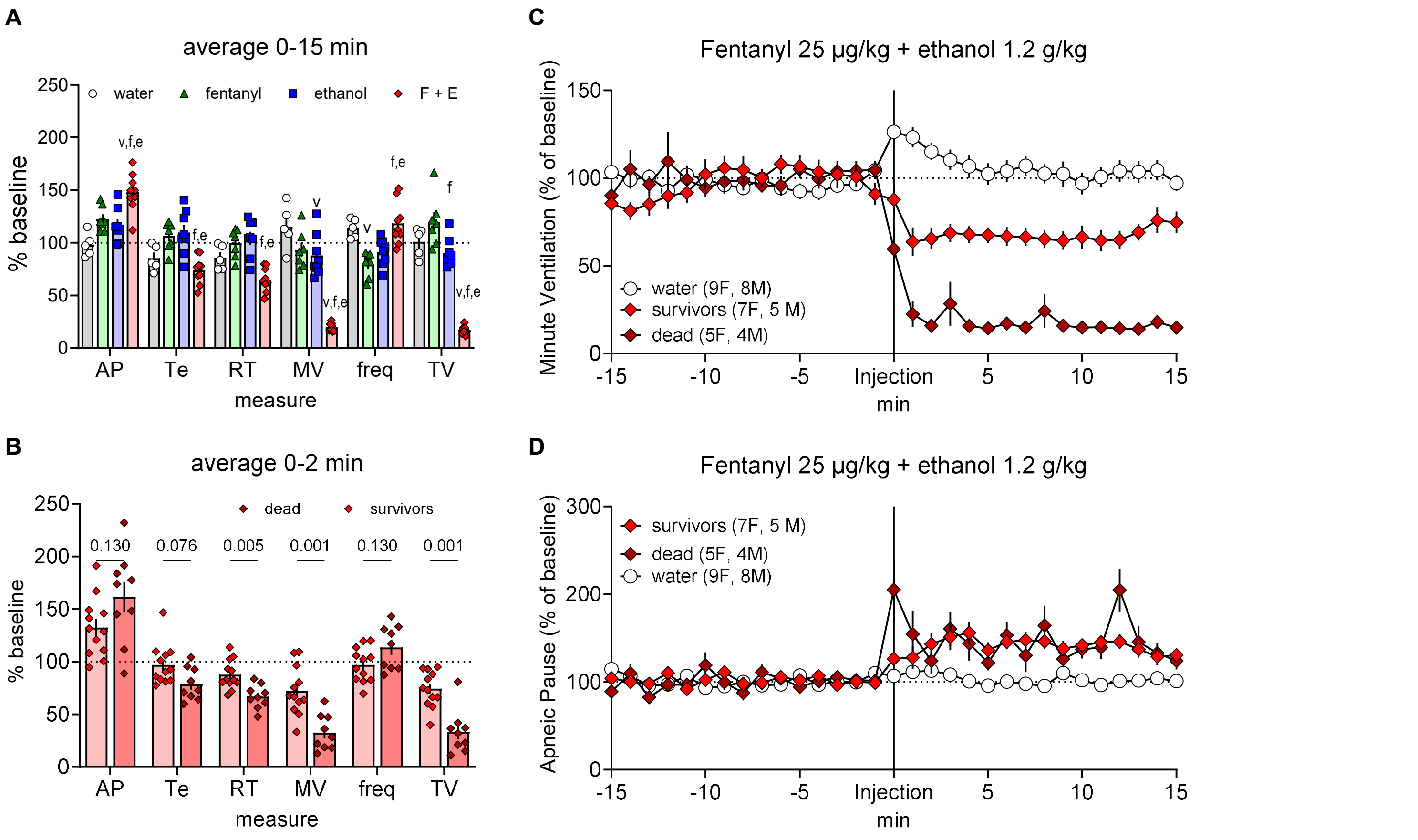

Supplemental Figure 2: Data from dead rats from Experiment 1. Rats received i.v. infusions of sterile water (2.5 ml/kg, 1 min), fentanyl (25 μg/kg, 5 ml/kg, 1 min), alcohol (30% v/v, 1.18 g/kg, 5 ml/kg, 1 min), or a

combination of fentanyl and alcohol (25 μg/kg and 1.18 g/kg, respectively, 5 ml/kg, 1 min) in a within-subjects Latin-square design with each test separated by 1 week. (**A**) Average of the first 15 min following vehicle, alcohol, fentanyl, and the combination of fentanyl and alcohol on apneic pause, expiratory time, relaxation time, minute ventilation, frequency and tidal volume. [f] different from fentanyl; [e] different from alcohol; [v] different from water ($p < 0.05$). (**B**) Average of the first 2 min following fentanyl and alcohol infusion in the surviving animals compared to the dead. (**C**) Time-dependent effect of the combination of fentanyl and alcohol (25 μg/kg and 1.18 g/kg, respectively, 5 ml/kg, 1 min) on minute ventilation. **(D**) Time-dependent effect of the combination of fentanyl and alcohol (25 μg/kg and 1.18 g/kg, respectively, 5 ml/kg, 1 min) on apneic pauses. Water group added for reference.

Experiment 2: 25 μg/kg fentanyl + 0.59 g/kg alcohol (2.5 mL/kg, "binge-like" dose)

This experiment recapitulated Experiment 1 but with only half of the alcohol dose, to achieve human binge-like BALs (>80 mg/dL), and n = 9-11/sex/drug. Each rat received a 1 min i.v. infusion of sterile water (2.5 mL/kg), fentanyl (25 μg/kg, 5 mL/kg), alcohol (0.59 g/kg, 2.5 mL/kg), or a combination of fentanyl and alcohol (25 μg/kg + 0.59 g/kg, 2.5 mL/kg) in a within-subjects Latin-square design. The rats weighed 241.9 ± 16.7 g (mean ± SD) and 206.5 ± 11.0 g (male and female, respectively) at the start of the study. By the end of the study, males gained an average of 180.2 g, and females gained an average of 83.3 g, and the mortality rate was 16.7% for females and 0% for males.

Experiment 3: 3.125 μg/kg fentanyl + 0.59 g/kg alcohol (2.5 mL/kg)

This experiment used the protocol of Experiment 1 to examine whether a dose of fentanyl that does not cause respiratory depression (i.e., a subeffective dose) potentiates respiratory effects of alcohol. Each rat received a 1 min i.v. infusion of sterile water (2.5 mL/kg), fentanyl (3.125 μg/kg, 2.5 mL/kg), alcohol (0.59 g/kg, 2.5 mL/kg), or a combination of fentanyl and alcohol (3.125 μg/kg + 0.59 g/kg, 2.5 mL/kg) in a within-subjects Latin-square design. The dose of fentanyl was based on a pilot

experiment that is shown in **Supplemental Figure 3**. The rats weighed 249.4 ± 13.1 g (mean ± SD) and 195.8 ± 22.0 g (male and female, respectively) at the start of the study. By the end of the study, males gained an average of 196.9 g, and females gained an average of 90.3 g.

To determine a subeffective dose of fentanyl we performed between-subjects plethysmography testing as described in the main manuscript. Following intravenous catheter implantation, rats were habituated for 1 h to the plethysmography chambers and given bolus intravenous injections of 0.1 mL flushing solution upon entering the chamber. Another 0.1 mL of flushing solution was administered through the swivel of the chamber prior to being removed the chamber. On the test day, the rats were placed in the chamber, and data acquisition began following a 10-min acclimation period. Baseline data was collected for 30 min, and then the rats received an intravenous bolus injection of sterile water or fentanyl (1.56, 3.125, 6.25, and 12.5 μg/kg, 1 ml/kg). Data were recorded for an additional 30 min post-injection.

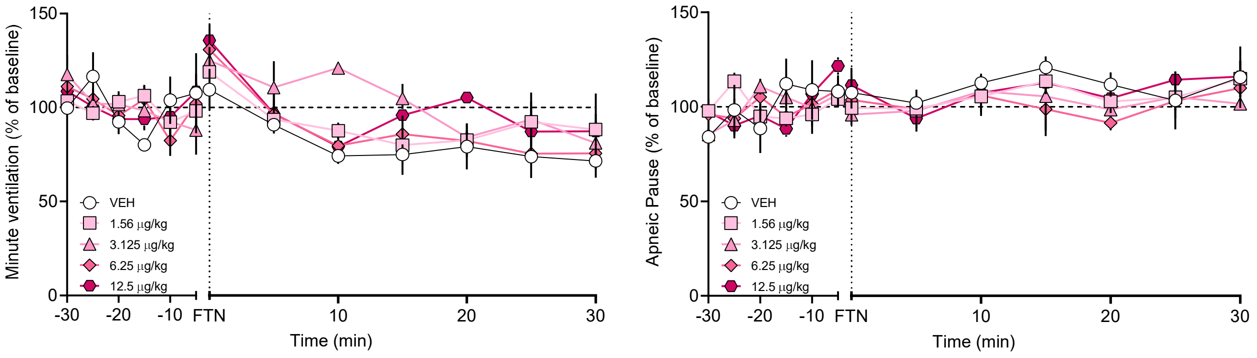

Supplemental Figure 3. Effect of low doses of fentanyl on minute ventilation and apneic pauses. Rats received i.v. infusions of sterile water (1 ml/kg) or fentanyl (1.56, 3.125, 6.25, and 12.5 μg/kg, 1 ml/kg), and breathing was monitored for 30 min (*n*=2-4/dose).

Following intravenous catheter implantation, rats were habituated for 1 h to the plethysmography chambers and given a bolus intravenous injection of 0.1 mL flushing solution upon entering the chamber. Another 0.1 mL of flushing solution was administered through the swivel of the chamber prior

to being removed the chamber. On the first test day, the rats were placed in the chamber, and data acquisition began following a 10-min acclimation period. Baseline data was collected for 30 min, and then the rats received an intravenous bolus injection of sterile water (2.5 ml/kg, 1 min), fentanyl (3 μg/kg, 2.5 ml/kg, 1 min), alcohol (30% v/v, 0.59 g/kg, 2.5 ml/kg, 1 min), or a combination of fentanyl and alcohol (3 μg/kg and 0.59 g/kg, respectively, 2.5 ml/kg, 1 min). Data were recorded for an additional 90 min post- injection. The same test was conducted over the course of 4 weeks until each rat received each dose in a within-subject Latin-square design. The area under the curve (AUC) was calculated for the first 15 min.

Analysis over time: The RM-ANOVA showed a main effect of time on minute ventilation ($F_{18,342}$=15.571, p<0.00001) and no effect of treatment ($F_{3,57}$=2.542, p=0.065) but a significant treatment × time interaction ($F_{54,1026}$=1.499, p=0.013; **Supplemental Figure 4A**). Alcohol decreased minute ventilation at 0-5 and 15 min post-infusion compared with vehicle (p<0.05), whereas fentanyl+alcohol decreased minute ventilation at 0 min post-infusion compared with water and at 5-10 and 30 min post-infusion compared with fentanyl alone (p<0.05). There was no significant effect of sex, no sex × treatment interaction, and no sex × treatment × time interaction. There was a significant sex × time interaction ($F_{18,342}$=1.842, p=0.02), with males and females differing at 5 and 10 min.

The RM-ANOVA showed a main effect of time on apneic pauses ($F_{18,342}$=6.361, p<0.00001) but no effect of treatment ($F_{3,57}$=0.493, p=0.688) and no treatment × time interaction ($F_{54,1026}$=1.175, p=0.186; **Supplemental Figure 4B**). There was no effect of sex, no sex × treatment interaction, no sex × time interaction, and no sex × treatment × time interaction.

Area under the curve: The AUC of minute ventilation for the first 15 min post-infusion showed a main effect of treatment ($F_{3,57}$=12.50, p<0.0001). Alcohol (p=0.0011) and fentanyl+alcohol (p=0.0007) but not fentanyl alone (p=0.99) induced a greater decrease in minute ventilation than vehicle. Fentanyl (p=0.0001) caused a lesser decrease in minute ventilation than fentanyl+alcohol

(**Supplemental Figure 4C**). There was a main effect of sex ($F_{1,19}$=7.114, p=0.015; females > males) but no sex × treatment interaction ($F_{3,57}$=0.509, p=0.677). The AUC of apneic pauses for the first 15 min post-infusion showed no main effect of treatment ($F_{3,57}$=2.083, p=0.113). There was no main effect of sex ($F_{1,19}$=2.244, p=0.151) and no sex × treatment interaction ($F_{3,57}$=0.887, p=0.453; **Supplemental Figure 4D**). Altogether, these data indicate additive effect of a binge-like dose of alcohol with a sub-effective dose of fentanyl on ventilatory parameters.

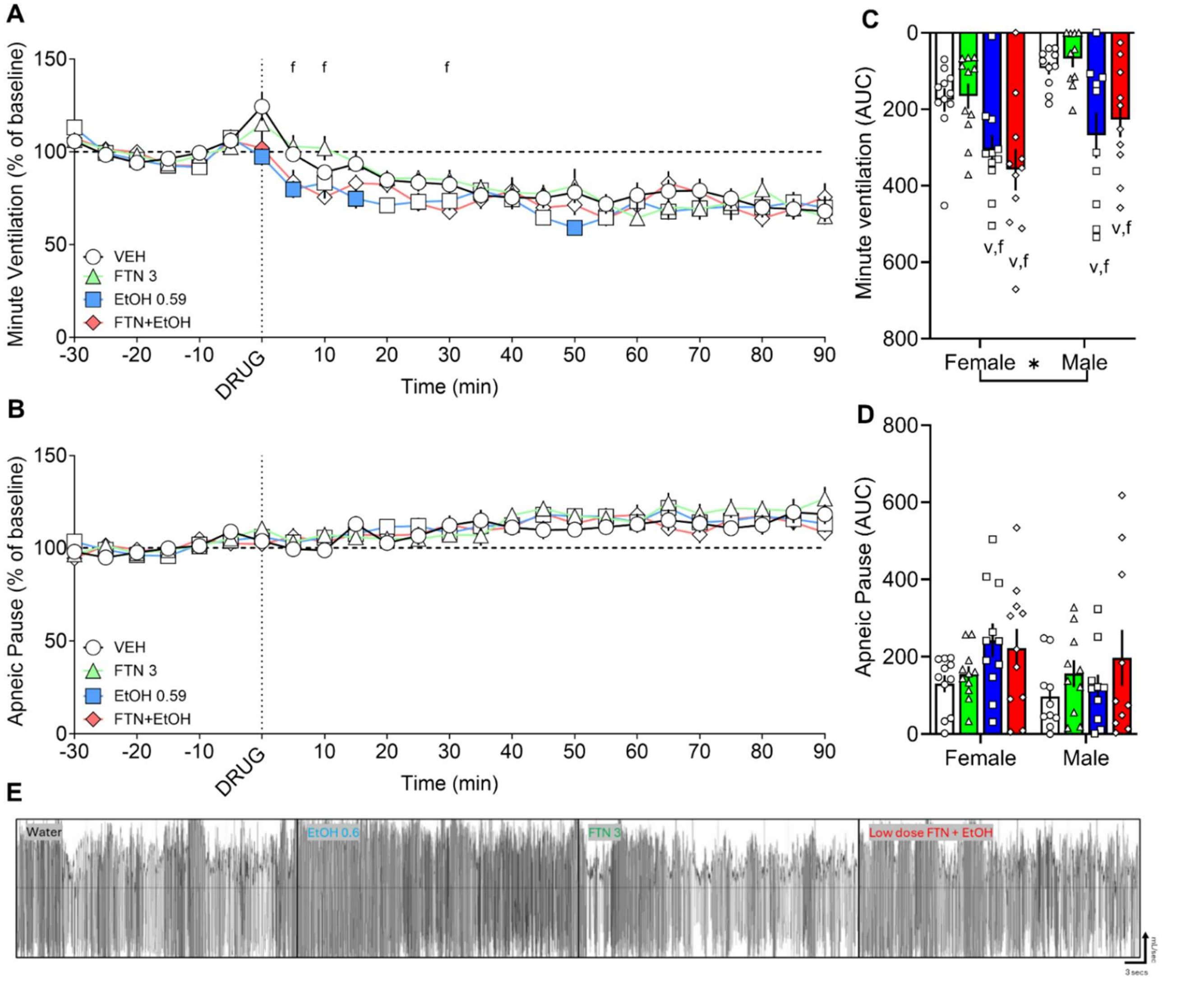

Supplemental Figure 4. Effects of 3 µg/kg fentanyl and 0.59 g/kg alcohol on minute ventilation and apneic pauses. Rats received i.v. infusions of sterile water (2.5 ml/kg, 1 min), fentanyl (3 µg/kg, 2.5 ml/kg, 1 min), alcohol (30% v/v, 0.59 g/kg, 2.5 ml/kg, 1 min), or a combination of fentanyl and alcohol (3 µg/kg and 0.59 g/kg, respectively, 2.5 ml/kg, 1 min) in a within-subjects Latin-square design with each test separated by 1 week. (**A**) Alcohol, fentanyl, and the combination of fentanyl and alcohol decreased minute ventilation in a time-dependent manner. (**B**) Alcohol, fentanyl, and the combination of fentanyl and alcohol increased apneic pauses in a time-dependent manner. The data are expressed as the mean ± SEM and were analyzed by two-way RM-ANOVA followed by Duncan's *post hoc* test when appropriate. Filled symbols are different from water. [f] Combination different from fentanyl; [e] combination different from

alcohol (p < 0.05). (**C**) Area under the curve of the first 15 min post-infusion for minute ventilation. (**D**) Area under the curve of the first 15 min post-infusion for apneic pauses. The data are expressed as the mean ± SEM and were analyzed by two-way RM-ANOVA followed by Šídák's *post hoc* test when appropriate. *p<0.05, compared with water; ^{f}p<0.05, compared with fentanyl; ^{e}p < 0.05, compared with alcohol. The data are expressed as the mean ± SEM and were analyzed by two-way RM-ANOVA followed by Šídák's *post hoc* test when appropriate. *p<0.05 compared with water; ^{f}p < 0.05, compared with fentanyl. n=11 female, 10 male. (**E**) Representative raw plethysmography traces.

Supplemental Table 1. Effects of 3 μg/kg fentanyl and 0.59 g/kg alcohol on ventilation measures.

**Area Under the Curve (15 min, calculated from 1 min bin data)**										
	Vehicle (2.5 mL/kg)		Fentanyl (3 μg/kg)		Alcohol (0.59 g/kg)		Fentanyl + Alcohol		ANOVA	
	F	M	F	M	F	M	F	M	Sex * Drug	Drug
**F**	125.3 (52.22)	30.48 (26.40)	115.8 (40.92)	23.74 (37.94)	183.9 (44.66)	22.63 (75.48)	255.0 (51.63)	111.0 (55.90)	$F_{3,76}$=0.245, p=0.8648	$F_{3,76}$=2.145, p=0.10
**TV**	4.831 (9.415)	11.84 (14.03)	00.00 (00.00)	6.547 (12.68)	102.8 (43.44)	172.5 (34.15)	104.4 (36.81)	65.08 (21.32)	$F_{3,76}$ =1.47, p=0.230	**$F_{3,76}$ = 12.21, p<0.0001**
**Ti**	44.77 (35.26)	3.883 (15.99)	112.1 (53.22)	73.39 (42.41)	238.0 (64.00)	172.1 (61.45)	356.6 (57.93)	167.0 (56.50)	$F_{3,56}$=0.936, p=0.43	**$F_{3,56}$ = 8.399, p=0.0001**
**Te**	123.1 (62.09)	46.69 (42.71)	113.1 (57.29)	22.66 (40.62)	213.8 (73.13)	108.7 (72.07)	500.9 (93.11)	193.8 (67.73)	$F_{3,64}$=1.418, p=0.25	**$F_{3,64}$ = 7.834, p=0.0002**
**PIF**	54.63 (39.01)	10.09 (12.13)	41.73 (44.91)	18.58 (23.52)	282.3 (52.06)	174.7 (73.91)	304.1 (63.87)	153.3 (57.49)	$F_{3,76}$=0.684, p=0.56	**$F_{3,76}$ = 10.40, p<0.0001**
**PEF**	121.0 (57.09)	20.44 (23.72)	86.04 (54.14)	00.00 (00.00)	270.6 (61.80)	122.4 (99.24)	303.6 (61.62)	193.20 (53.68)	$F_{3,76}$=0.103, p=0.96	**$F_{3,76}$ = 5.698, p=0.0014**
**RT**	131.1 (63.07)	82.05 (51.98)	161.3 (79.62)	17.72 (41.38)	189.8 (80.54)	106.1 (71.12)	338.5 (66.37)	154.6 (62.88)	$F_{3,64}$=0.431, p=0.73	$F_{3,64}$ = 2.350, p=0.08
**EIP**	2.596 (18.27)	15.38 (20.66)	5.959 (21.19)	65.94 (45.19)	419.5 (50.58)	389.0 (52.54)	435.1 (43.35)	387.7 (52.21)	$F_{3,72}$=0.701, p=0.55	**$F_{3,72}$ = 60.27, p<0.0001**
**EEP**	88.57 (49.69)	5.189 (19.83)	177.1 (73.67)	42.44 (40.86)	353.6 (78.22)	149.4 (59.45)	488.2 (77.83)	285.4 (81.17)	$F_{3,56}$=0.423, p=0.74	**$F_{3,56}$ = 11.43, p<0.0001**

The data are expressed as the mean (SEM). [V]Different from vehicle; [F]different from fentanyl; [E]different from alcohol.

Experiment 4: naloxone reversal of respiratory depression induced by 25 μg/kg fentanyl + 0.59 g/kg alcohol

Using the same rats from Experiment 3 (*n* = 5 males, 10 females) and nondependent rats from Experiment 5 (*n* = 3/sex), we tested whether 100 μg/kg naloxone reversed respiratory depression that was caused by the combination of 0.59 g/kg alcohol and 25 μg/kg fentanyl. Each rat received a 1 min i.v. infusion of a combination of fentanyl and alcohol (25 μg/kg + 0.59 g/kg, 2.5 mL/kg). Five minutes later, the rats received a bolus injection of naloxone (0 and 100 μg/kg, 1 mL/kg, i.v.). Ventilation was monitored for 90 min after naloxone administration. The same procedure was repeated the following week so that the rats received both vehicle and naloxone. The rats weighed 249.4 ± 13.1 g (mean ± SD) and 195.8 ± 22.0 g (males and females, respectively) at the start of the study. By the end of the study, males gained an average of 239.7 g, and females gained an average of 103.5 g.

The rats from Experiment 3 were housed on a different light cycle than the rats from Experiment 5. To avoid stressing the animals and potentially losing catheter patency during a week-long transition, we did not change the light cycle of either group of rats before testing the ability of naloxone to reverse respiratory depression. Once the tests were complete, we statistically compared the data from male rats that were on a regular light cycle (lights on at 6 AM; previously completed Experiment 3) and from male rats that were on a reverse light cycle (lights on at 8 PM; previously completed Experiment 5). We found no differences in any parameter between the two groups (data not shown).

A separate cohort of rats was used to test whether higher doses of naloxone reverse respiratory depression that is caused by the combination of 0.59 g/kg alcohol and 25 μg/kg fentanyl. Each rat received a 1 min i.v. infusion of a combination of fentanyl and alcohol (25 μg/kg + 0.59 g/kg, 2.5 mL/kg). Five minutes later, the rats received a bolus injection of naloxone (0, 300, and 1000 μg/kg, 1 mL/kg, i.v.). Ventilation was monitored for 90 min following naloxone administration. The same procedure was repeated the following 2 weeks so that the rats received both doses of naloxone and vehicle. The rats weighed 212.4 ± 33.4 g (mean ± SD) and 181 ± 15.7 g (males and females, respectively) at the start of the study. By the end of the study, males gained an average of 124.4 g, and females gained an average of 69.9 g.

Lastly, we tested whether 300 μg/kg naloxone (1 mL/kg, i.v.) prevents effects of a combination of fentanyl and alcohol (25 μg/kg + 0.59 g/kg, 2.5 mL/kg, i.v.). During testing, the rats were acclimated to the chambers for 10 min, followed by 30 min of baseline collection. Each rat received a bolus injection of naloxone (0 or 300 μg/kg, 1 mL/kg, i.v.), followed by a 1 min i.v. infusion of a combination of fentanyl and alcohol (25 μg/kg + 0.59 g/kg, 2.5 mL/kg) 5 min later. Ventilation was then monitored for 90 min post-administration. The same procedure was repeated the following week so that the rats received both vehicle and naloxone. The rats weighed 256.9 ± 14.3 g (mean ± SD) and 202.3 ± 19.0 g (males and females, respectively) at surgery. By the end of the study, males gained an average of 61 g, and females gained an average of 42.9 g.

Naloxone reverses respiratory depression induced by 25 μg/kg fentanyl or 0.59 g/kg alcohol

A separate cohort of rats was used to test whether naloxone 300 μg/kg reverses respiratory depression that is caused by 0.59 g/kg alcohol or 25 μg/kg fentanyl. Each rat received

a 1 min i.v. infusion alcohol (0.59 g/kg, 2.5 mL/kg) or a bolus injection of fentanyl (25 µg/kg, 1 mL/kg). Five minutes later, the rats received a bolus injection of naloxone (0 or 300 µg/kg, 1 mL/kg, i.v.). Ventilation was monitored for 60 min following naloxone administration. The same procedure was repeated the following week so that the rats received both naloxone and vehicle. Alcohol caused only mild ventilatory effects that were not modified by naloxone (**Supplemental Figures 5 and 6**). Fentanyl led to respiratory depression that was reversed by naloxone 300 µg/kg (**Supplemental Figures 5 and 7**).

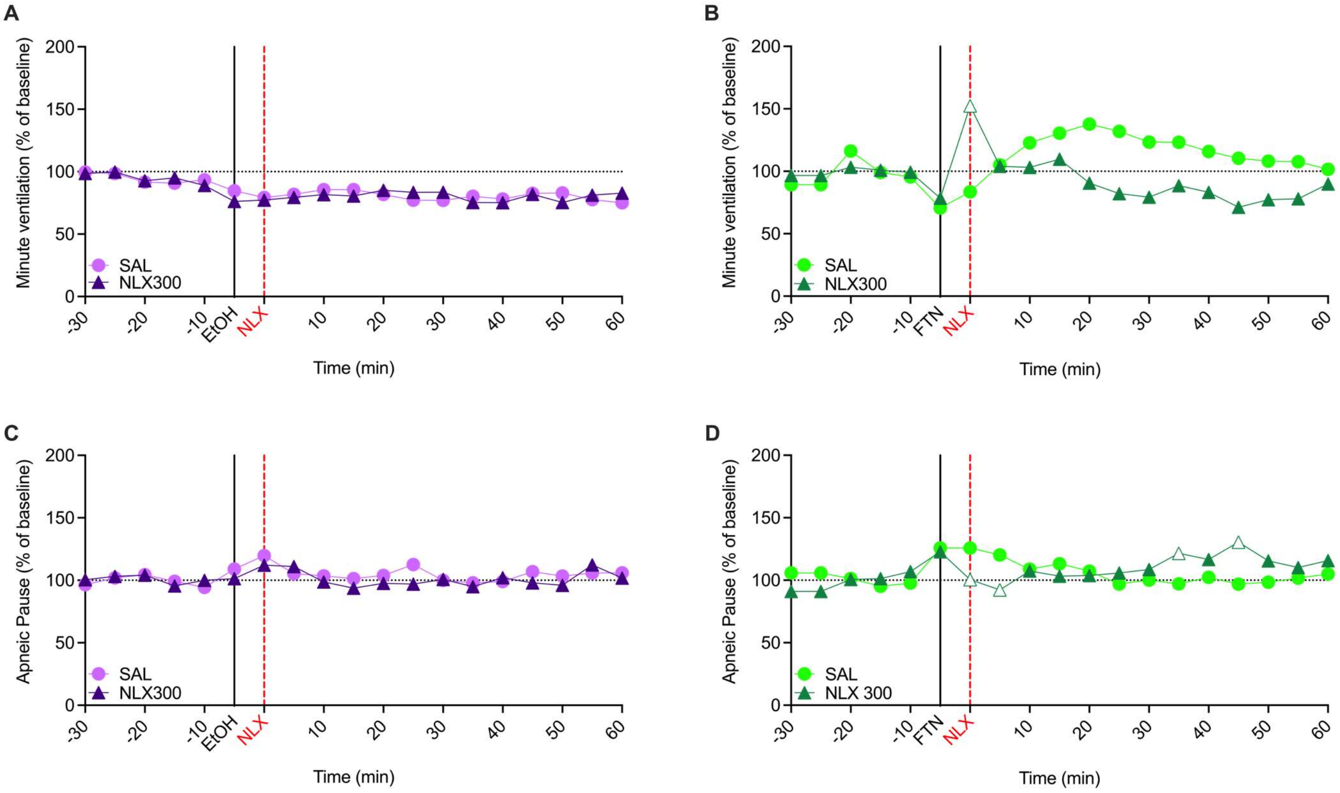

Supplemental Figure 5. Effect of naloxone on the respiratory depression caused by fentanyl or alcohol. Rats received an i.v. infusion of fentanyl (25 µg/kg, 1 ml/kg) (**B and D**) or alcohol (0.59 g/kg, 2.5 ml/kg, 1 min) (**A and C**) followed by an injection of naloxone (0 or 300 µg/kg) 5 min later. The data are expressed as the mean ± SEM and were analyzed by two-way RM-ANOVA followed by Duncan's *post*

hoc test when appropriate. Open symbols indicate a treatment effect at each timepoint. *n*=15-16, 8 males, 7-8 females.

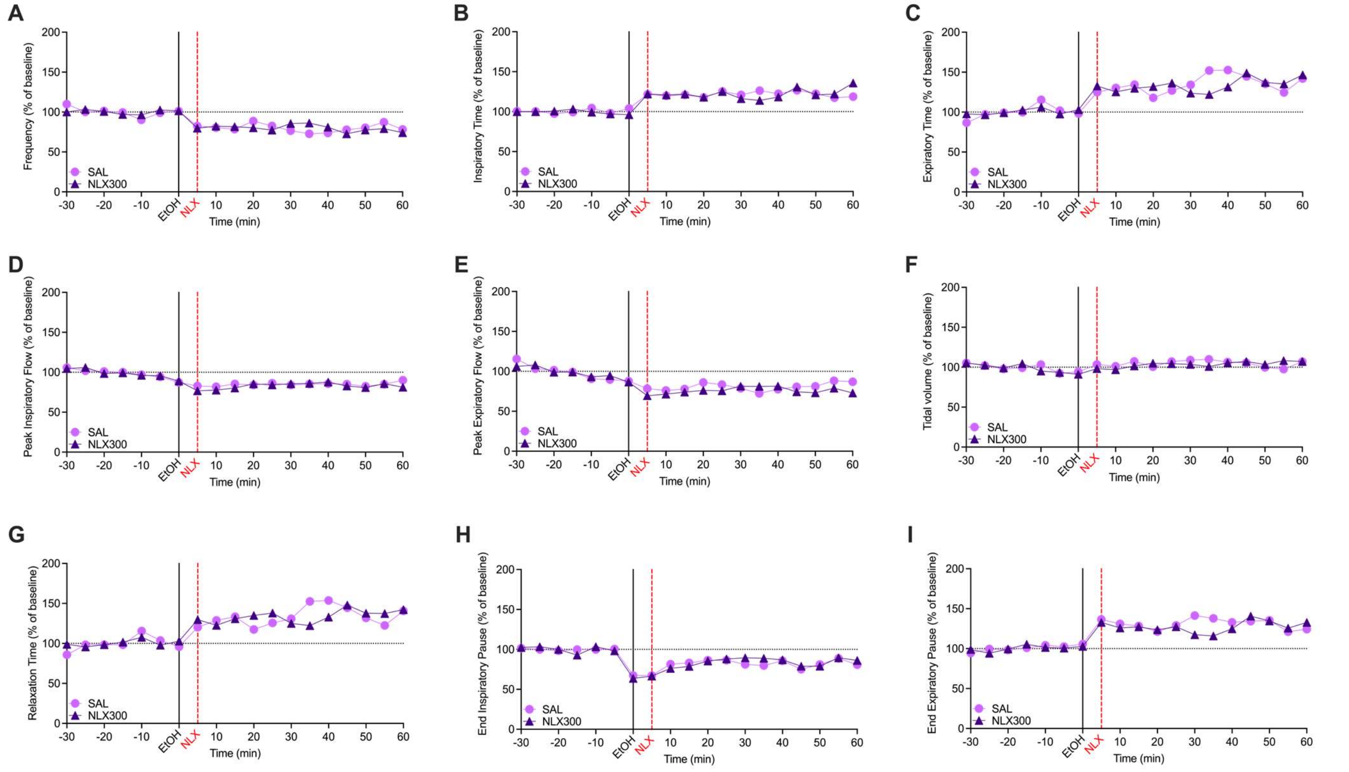

Supplemental Figure 6. Effect of naloxone on the respiratory depression caused by alcohol. Rats received an i.v. infusion of alcohol (0.59 g/kg, 2.5 ml/kg, 1 min) followed by an injection of naloxone (0 or 300 μg/kg) 5 min later. The data are expressed as the mean ± SEM and were analyzed by two-way RM-ANOVA followed by Duncan's *post hoc* test when appropriate. Open symbols indicate a treatment effect at each timepoint. *n*=16, 8 males, 8 females.

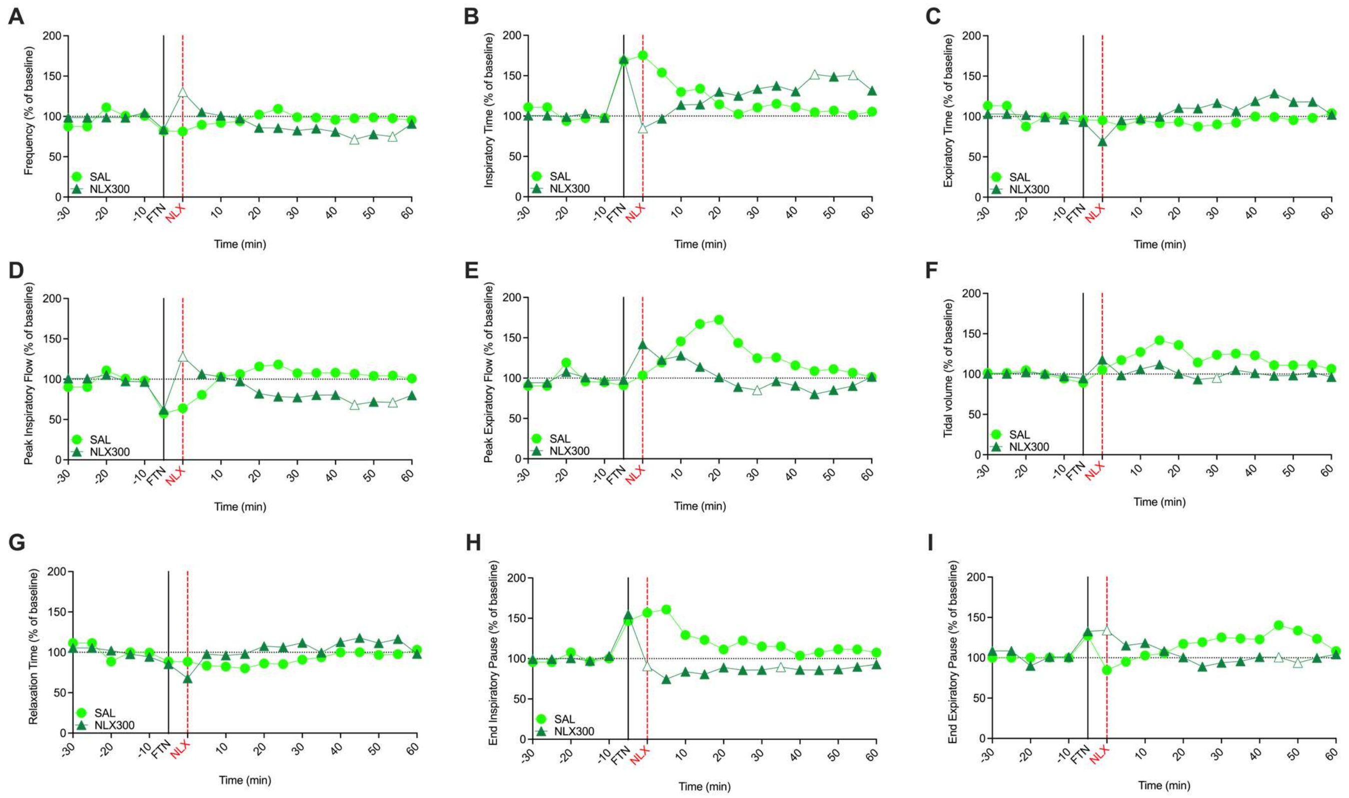

Supplemental Figure 7. Effect of naloxone on the respiratory depression caused by fentanyl. Rats received an i.v. infusion of fentanyl (25 µg/kg, 1 ml/kg) followed by an injection of naloxone (0 or 300 µg/kg) 5 min later. The data are expressed as the mean ± SEM and were analyzed by two-way RM-ANOVA followed by Duncan's *post hoc* test when appropriate. Open symbols indicate a treatment effect at each timepoint. *n*=15, 8 males, 7 females.

Experiment 5: 25 µg/kg fentanyl + 0.59 g/kg alcohol in fentanyl- and alcohol-dependent rats

Rats (eight males, seven females) were made dependent on alcohol as described above. Fentanyl-dependent rats were tested 4-6 h into withdrawal. Nondependent rats (eight male, seven female) were concomitantly tested. All rats were catheterized during the same week and habituated to the plethysmography chambers 1 week later following the protocol that is described in Experiment 1. Each rat received a 1 min i.v. infusion of fentanyl (25 µg/kg, 2.5

mL/kg), alcohol (0.59 g/kg, 2.5 mL/kg), or a combination of fentanyl and alcohol (25 µg/kg + 0.59 g/kg, 2.5 mL/kg) in this order at 1-week intervals between tests. If a rat's catheter was not patent on a testing day, then it was either repaired or replaced, and the drug was re-administered in a makeup test in the following week. The rats weighed 197.1 ± 17.3 g (mean ± SD) and 160.8 ± 17.3 g (male and female, respectively) at the start of the study. By the end of the study, the alcohol-dependent males and females gained an average of 154.9 and 77.2 g, the fentanyl-dependent males and females gained an average of 169.4 and 85.7 g, and the nondependent males and females gained an average of 135.6 and 82.5 g, respectively.

We compared the ventilatory response at baseline for all groups and found no statistical differences between the groups in any of the 11 parameters measured. The bottom panel show representative plethysmography traces for each group following treatment with alcohol 1.18 g/kg (EtOH), fentanyl 25 µg/kg (FTN) or the combination of alcohol 1.18 g/kg + fentanyl 25 µg/kg (FTN+EtOH).

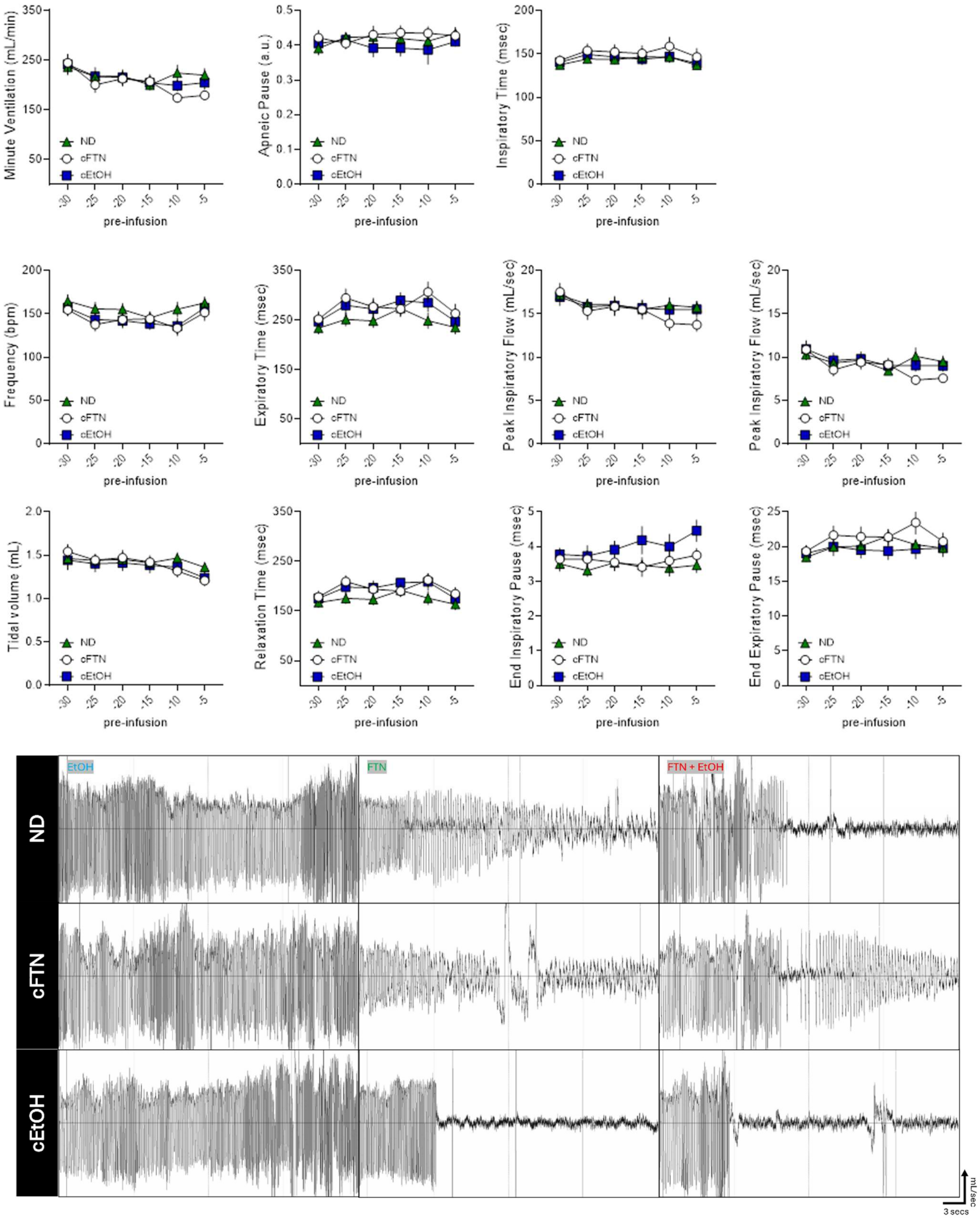
Minute Ventilation (mL/min)
Apneic Pause (a.u.)
Inspiratory Time (msec)
Frequency (bpm)
Expiratory Time (msec)
Peak Inspiratory Flow (mL/sec)
Peak Inspiratory Flow (mL/sec)
Tidal volume (mL)
Relaxation Time (msec)
End Inspiratory Pause (msec)
End Expiratory Pause (msec)
ND
cFTN
cEtOH
pre-infusion
-30
-25
-20
-15
-10
-5
EtOH
FTN
FTN + EtOH
mL/sec
3 secs

Supplemental Figure 8. There is no effect of chronic exposure to alcohol or fentanyl on baseline breathing parameters. The data are expressed as the mean ± SEM and were analyzed by two-way RM-ANOVA followed by Duncan's *post hoc* test when appropriate. *n* = 11-13, 5-8 males, 5-8 females. Bottom: Representative raw plethysmography traces.

Experiment 6: blood gasometry and blood and brain drug concentration measurements

Twenty rats (10 male, 10 female) with jugular vein and femoral artery catheters were used. One week before this experiment, these rats underwent a test to examine the effect of an investigational compound on heroin-induced respiratory depression. Based on previous experiments with this compound, one week was enough time for full washout. In Experiment 6, the rats received a 1 min i.v. infusion of fentanyl (25 µg/kg, 2.5 mL/kg), alcohol (0.59 g/kg, 2.5 mL/kg), or a combination of fentanyl and alcohol (25 µg/kg + 0.59 g/kg, 2.5 mL/kg; *n* = 5/group, 2-3 females). Five minutes following the infusion, arterial blood was collected for the measurement of blood gases. The rats were then anesthetized (2-3% isoflurane in O_2) and euthanized 10 min post-infusion. Trunk blood and brains were collected, flash-frozen using 2-methylbutane (Sigma-Aldrich, St. Louis, MO, USA) on dry ice and then stored at -80°C until the analysis of fentanyl and alcohol levels. The rats weighed 299.6 ± 12 g (mean ± SD) and 211.4 ± 10.5 g (males and females, respectively).

To measure blood gas levels, 100 µl of arterial blood was injected into CG8+ cartridges (Abbott Laboratories, Chicago, IL, USA) and analyzed using an i-STAT 1 analyzer (Abbott Laboratories). Measurements of eight variables were obtained (**Table 3**).

Brain tissue homogenization

Brain tissue was weighed, and 4 mL of water per gram of tissue was added to tubes that contained ceramic beads. The samples were homogenized using a Bead Ruptor bead mill homogenizer (Omni International, Kennesaw, GA, USA).

Fentanyl and norfentanyl measurements

Fentanyl and norfentanyl analytical standards (Cerilliant, Round Rock, TX, USA) were used to spike calibrators in blank serum from 0.25 to 10 ng/mL or in blank brain tissue homogenates from 0.4 to 40 pg/mg. Twenty (serum) or 100 (brain tissue) microliters of the calibrators or unknown samples were protein precipitated with 60 µL (serum) or 400 µL (brain tissue) of acetonitrile. Next, 10 µl of 10 ng/mL deuterated fentanyl and norfentanyl (Cerilliant, Round Rock, TX, USA) was added to each calibrator and unknown samples for normalization. The calibrators and samples were shaken at 2,000 rotations per minute for 5 min and centrifuged at 20,000 × *g* for 5 min at 4 °C. Fifty (serum) or 100 (brain tissue) microliters of the supernatant were transferred to a glass autosampler vial from which 10 µl was injected into the liquid chromatography-mass spectrometry instrument.

Sample analysis was performed using a Vanquish UHPLC system (ThermoFisher, Waltham, MA, USA) coupled to an Orbitrap Exploris 120 mass spectrometer (ThermoFisher). Reverse-phase chromatography was performed using an Accucore Biphenyl column, 2.1 × 50 mm, 2.6 µm particle size column (ThermoFisher), with 10 mM ammonium formate in 0.1% formic acid as mobile phase A and methanol as mobile phase B. The flow rate was 0.4 mL/min, and the solvent gradient for mobile phase B was the following: 0-0.5 min held at 10%, 0.5-3.0 min increased from 5% to 95%, 3-5 min held at 95%, 5-6 min decreased from 95% to 10%, and then held at 10% to 7.5 min. The analysis was performed in positive ion mode with a

full scan mass range of 200-350 *m/z* and a mass resolution of 120K. Ionization was conducted using a heated electron spray ionization source. XCalibur 4.4.16.14 software (ThermoFisher) was used to integrate and report the peak area for M+H ions (norfentanyl: 233.1648 *m/z* at a retention time of 3.45 min; fentanyl: 337.2274 *m/z* at a retention time of 3.90 min) and to plot and fit a standard curve and interpolate unknown values.

Blood alcohol measurements

An alcohol calibration curve was prepared from 12.5-300 mg/dL (serum) and 5-400 mg/dL (brain tissue) using alcohol standards (Cerilliant, Round Rock, TX, USA) in water. Briefly, 10 μL of alcohol standard or serum and 100 μL of alcohol standard or brain tissue homogenate was added to 10 mL glass headspace vials (Agilent, Santa Clara, CA, USA) and sealed with a crimp cap. The vials were heated in a 70°C 7697 A Headspace Sampler (Agilent) before headspace injection onto an MXT-Volatiles 30m, 0.28 mm inner diameter, 1.25 μm df column (Restek, Center County, PA, USA) using helium as the carrier gas. The 8890 gas-chromatography system column oven (Agilent) was heated to 40°C for an isocratic 6 min run that was paired with a 5977B gas-chromatograph/mass selective detector (Agilent). Alcohol concentrations were measured in positive ion mode with an electron impact source at 31.1 *m/z* as the quantifier and 45.1 and 46.1 *m/z* as the qualifiers.

Statistical analysis

A custom-made application (rvent_app) was used to import, bin, compile, plot, and export Microsoft Excel datasheets from the .txt files that were generated and calculated using iox 2.10.0.40 software (emka TECHNOLOGIES, Paris, France). Prism 8 software (GraphPad,

San Diego, CA, USA) was used for figure preparation. Statistica 13 software (TIBCO, Palo Alto, CA, USA) was used for statistical analyses. All data were aggregated into 1-min bins for analysis of the first 15 min post-infusion or in 5-min bins for the whole 90 min session analyses. The data are expressed as the mean ± SEM percentage of baseline values. Baseline values were calculated from the average pre-drug baseline values that were recorded between -30 to -5 min for each individual rat ($100 \times \frac{Value}{Baseline}$). Outliers were identified in Prism using the ROUT test with the maximum desired false discovery rate (Q) set to 1%. Excluded outliers were replaced by the average of temporally proximal values. The whole dataset of an individual rat for a given variable was excluded if > 25% of the data post-drug infusion were identified as outliers. The exclusion criterion was applied to each respiratory variable separately.

The 1-min data of the first 15 min post-infusion were used to calculate the area under the curve. The positive peak area and maximum value of each rat were used for variables where respiratory depression was characterized by an increase (i.e., inspiratory and expiratory times, apneic pause, end-inspiratory-pause, and end-expiratory-pause). The negative peak area and minimum value of each rat were used for variables where respiratory depression was characterized by a decrease (i.e., minute ventilation, frequency, tidal volume, peak inspiratory flow, and peak expiratory flow). The total peak area for all variables was used in naloxone trials.

For statistical purposes, the analyses of treatment over time included the post-injection data but not the baseline data, we used three-way repeated-measures analysis of variance (RM-ANOVA), with drug (treatment) and time as within-subjects factors and sex as a between-subjects factor. To determine the effect of treatments on the area under the curve, we used

two-way RM-ANOVA, with sex as a between-subjects factor and drug (treatment) as a within-subjects factor. Dunnett, Duncan, and Šídák *post hoc* tests were used when appropriate. Values of $p < 0.05$ were considered statistically significant.

REFERENCES

1. Levine B, Green D, Smialek JE. The role of ethanol in heroin deaths. *J Forensic Sci*. 1995;40(5):808–810.

2. Vendruscolo LF, et al. Escalation patterns of varying periods of heroin access. *Pharmacology Biochemistry and Behavior*. 2011;98(4):570–574.

3. Marchette RCN, et al. Heroin- and Fentanyl-Induced Respiratory Depression in a Rat Plethysmography Model: Potency, Tolerance, and Sex Differences. *J Pharmacol Exp Ther*. 2023;385(2):117–134.

4. Wade CL, et al. Compulsive-Like Responding for Opioid Analgesics in Rats with Extended Access. *Neuropsychopharmacol*. 2015;40(2):421–428.

5. Priddy BM, et al. Sex, strain, and estrous cycle influences on alcohol drinking in rats. *Pharmacol Biochem Behav*. 2017;152:61–67.

6. Vendruscolo LF, et al. Corticosteroid-dependent plasticity mediates compulsive alcohol drinking in rats. *J Neurosci*. 2012;32(22):7563–7571.

7. Vendruscolo LF, et al. Glucocorticoid receptor antagonism decreases alcohol seeking in alcohol-dependent individuals. *J Clin Invest*. 2015;125(8):3193–3197.

8. Gilpin NW, et al. Operant behavior and alcohol levels in blood and brain of alcohol-dependent rats. *Alcohol Clin Exp Res*. 2009;33(12):2113–2123.

9. Vendruscolo LF, Roberts AJ. Operant alcohol self-administration in dependent rats: focus on the vapor model. *Alcohol*. 2014;48(3):277–286.

10. Edwards S, et al. Divergent regulation of distinct glucocorticoid systems in alcohol dependence. *Alcohol*. 2015;49(8):811–816.
